# HMA-Net: a hybrid mixer framework with multihead attention for breast ultrasound image segmentation

**DOI:** 10.3389/frai.2025.1572433

**Published:** 2025-06-18

**Authors:** Soumya Sara Koshy, L. Jani Anbarasi

**Affiliations:** School of Computer Science and Engineering, Vellore Institute of Technology, Chennai, India

**Keywords:** breast cancer, deep learning, ultrasound images, segmentation, ConvNeXt, ConvMixer

## Abstract

**Introduction:**

Breast cancer is a severe illness predominantly affecting women, and in most cases, it leads to loss of life if left undetected. Early detection can significantly reduce the mortality rate associated with breast cancer. Ultrasound imaging has been widely used for effectively detecting the disease, and segmenting breast ultrasound images aid in the identification and localization of tumors, thereby enhancing disease detection accuracy. Numerous computer-aided methods have been proposed for the segmentation of breast ultrasound images.

**Methods:**

A deep learning-based architecture utilizing a ConvMixer-based encoder and ConvNeXT-based decoder coupled with convolution-enhanced multihead attention has been proposed for segmenting breast ultrasound images. The enhanced ConvMixer modules utilize spatial filtering and channel-wise integration to efficiently capture local and global contextual features, enhancing feature relevance and thus increasing segmentation accuracy through dynamic channel recalibration and residual connections. The bottleneck with the attention mechanism enhances segmentation by utilizing multihead attention to capture long-range dependencies, thus enabling the model to focus on relevant features across distinct regions. The enhanced ConvNeXT modules with squeeze and excitation utilize depthwise convolution for efficient spatial filtering, layer normalization for stabilizing training, and residual connections to ensure the preservation of relevant features for accurate segmentation. A combined loss function, integrating binary cross entropy and dice loss, is used to train the model.

**Results:**

The proposed model has an exceptional performance in segmenting intricate structures, as confirmed by comprehensive experiments conducted on two datasets, namely the breast ultrasound image dataset (BUSI) dataset and the BrEaST dataset of breast ultrasound images. The model achieved a Jaccard index of 98.04% and 94.84% and a Dice similarity coefficient of 99.01% and 97.35% on the BUSI and BrEaST datasets, respectively.

**Discussion:**

The ConvMixer and ConvNeXT modules are integrated with convolution-enhanced multihead attention, which enhances the model's ability to capture local and global contextual information. The strong performance of the model on the BUSI and BrEaST datasets demonstrates the robustness and generalization capability of the model.

## 1 Introduction

Breast cancer is the most frequently diagnosed and deadliest form of cancer, primarily affecting women. Breast cancer is caused by the irregular growth of abnormal cells in the breast, leading to tumors. Tumors can be classified as either benign or malignant. Benign tumors are non-cancerous and do not spread outside the breast tissues, while malignant tumors are cancerous and have the ability to metastasize beyond the breast tissues to other parts of the body. Effective policies and initiatives have reduced the percentage of women with metastatic breast cancer at diagnosis in high-income nations; nonetheless, disparity still exists, which must be addressed by raising awareness of breast cancer symptoms and promoting early detection (Fuentes et al., 2024). In economically deprived nations, the occurrence and fatality rates of breast cancer continue to increase. Early detection can significantly reduce breast cancer mortality. Research pertaining to the detection of the disease at an early stage has gained wide attention.

Due to the non-invasive, real-time, low-cost, and non-radiation nature of ultrasound imaging, it has quickly gained widespread acceptance as a breast tumor detection method. Ultrasound imaging uses sound waves instead of radiation to generate images. A precise analysis of the breast cancer images can be done by extracting only the relevant areas from the images, which is called segmentation. Breast ultrasound lesion segmentation presents a variety of challenges: (1) Poor contrast and noise present in the images make it difficult to differentiate lesions from the surrounding tissues. (2) Variations in the structure of malignant tumors make them difficult to detect. (3) Uneven distribution of benign and malignant images in the datasets.

The manual annotation of breast ultrasound images is laborious, time-intensive, and prone to inter-observer variability; an automated segmentation approach can mitigate these issues by delivering consistent and trustworthy outcomes. Computer-aided diagnostic tools for breast ultrasound images have steadily gained popularity as a means of enhancing the precision of diagnosis. Precise segmentation can augment diagnostic accuracy, facilitate quantitative lesion analysis, and aid radiologists in making more informed judgments, thereby enhancing patient outcomes. Furthermore, automating segmentation can optimize clinical procedures, decrease diagnostic duration, and facilitate radiology training by offering prompt feedback. Earlier machine-learning techniques were utilized for breast ultrasound image segmentation, for which manual intervention was required for feature extraction. This approach is time-consuming and also lacks consistency and reliability. Deep learning methods have now been widely used for breast ultrasound image segmentation, in which features are extracted automatically. Various convolutional neural network architectures, including U-Net, U-Net++, etc., show exceptional performance in ultrasound image segmentation.

A hybrid mixer framework with multihead attention (HMA-Net) is proposed for breast ultrasound image segmentation in which features are extracted from the input ultrasound images using five contiguous ConvMixer (Trockman and Kolter, 2022)-based encoder blocks (*EM*_*x*_*i*__), which utilize enhanced ConvMixer for improved feature representation across channels. Convolution-enhanced multihead attention (*CE*_*MHA*_) acts as an intermediary between the encoder and decoder, extracting significant semantic information and effectively decreasing the number of channels, thereby reducing the computational complexity of subsequent layers. Multihead attention (Georgescu et al., 2023) enables the model to focus on divergent areas of the image simultaneously, thereby allowing the model to capture intricate patterns. The ConvNeXT-based decoder blocks (*DCN*_*x*_*i*__) generate high-resolution feature maps from the compressed feature maps produced by the *EM*_*x*_*i*__ blocks. The ConvNeXT (Liu et al., 2022) involves enhanced convolutions with which the features can be extracted with increased efficiency compared to the conventional convolutional networks. Skip connections are established between the feature maps in the contracting path of the *EM*_*x*_*i*__ blocks and the corresponding layers in the *DCN*_*x*_*i*__, facilitating feature merging *via* concatenation to restore the spatial resolution of images.

The HMA-Net model is validated on two datasets of ultrasound images. The initial dataset is called breast ultrasound image dataset (BUSI) (Al-Dhabyani et al., 2020), which consists of 780 ultrasound images in PNG format. The second dataset is a benchmark dataset, called BrEaST (Pawłowska et al., 2024), of ultrasound images. It consists of 256 breast images from 256 patients, all of which have been personally annotated by a skilled radiologist.

The major contributions of this study are as follows:

(1) ConvMixer-based encoders for efficiently extracting and summarizing the features of input images.
(2) ConvNeXT-based decoders for efficiently reconstructing feature maps based upon the intricate features received from the encoder.
(3) Channel-wise feature responses of each channel are recalibrated using the squeeze and excitation by explicitly modeling the interdependencies among channels.
(4) A computationally efficient bottleneck, combined with convolution-enhanced multihead attention, allows for the simultaneous processing of multiple components of the input sequence, capturing their intricate relationships.
(5) The encoder, decoder, and enhanced multihead attention utilize residual connections to combine high-level and low-level features. It facilitates stable and faster training by diminishing the problem of vanishing gradients.
(6) Utilization of the combined loss function (Adrian et al., 2022) enhances the ability of the model to deal with unbalanced data.

The subsequent sections are organized as follows: Section II presents a summary of the recent research in the field of breast ultrasound lesion segmentation. The architecture of the HMA-Net is elaborated in Section III. Section IV covers experimental results and discussions. Section V discusses the conclusion.

## 2 Related works

Researchers have extensively studied breast ultrasound image segmentation, which is the primary step in breast cancer detection. Conventional approaches used thresholding-based methods (Horsch et al., 2001), watershed-based methods (Huang and Chen, 2004), clustering-based methods (Moon et al., 2014), graph-based methods (Zhou et al., 2014), etc., for segmentation. Recently, researchers have utilized deep learning methods based on convolutional neural networks and proposed various approaches for breast ultrasound image segmentation. Üzen (2024) introduced an encoder–decoder network in which the encoder is based on ConvMixer, and the decoder utilizes classification techniques. DenseNet121 is used in the encoder part to obtain semantic and spatial information, whereas long-range contextual information is acquired with ConvMixer. The encoder merges and passes the features to the decoder, which employs a detection and classification network to obtain the classification and detection scores. The performance of the approach is analyzed using the BUSI dataset.

Zhang et al. (2023) introduced a method that includes a classification branch and a segmentation branch. The classification branch receives the encoder's output and classifies the images into normal and abnormal. The classification branch is responsible for determining whether the image is benign or cancerous, and the segmentation branch draws the outlines of the tumors. A new breast ultrasound dataset has been compiled with 1,600 images, 405 of which were benign, 372 were malignant, and the rest were normal. Xu et al. (2023) proposed a regional attentive multitask learning framework for classifying and segmenting breast ultrasound images. A regional attention module was designed in which predicted probability maps are utilized to direct the classifier to learn category-specific information in the background, peritumoral, and tumor regions, which are then combined to enhance the feature representation. The model involves a segmentation and classification network that shares the features acquired from the encoder. This study used the BUSI and UDIAT datasets.

Chen et al. (2023) presented a method in which a deeper U-Net is employed to capture feature information from ultrasound images. Between the encoder and decoder, the squeeze and excitation network, acts as a link to enhance attention. Prediction masks of the ultrasound images are refined by incorporating deep supervised constraints to the decoding network. The method is analyzed using two datasets: BUSI and Dataset B (Yap et al., 2017). Lyu et al. (2023) combined attention mechanisms and multiscale features for segmenting breast ultrasound images. The authors performed multidimensional feature extraction using a depthwise separable convolution strategy on the encoding side and utilized Global Attention Upsample feature fusion on the decoding side. The model is evaluated using two datasets (Al-Dhabyani et al., 2020; Piotrzkowska-Wróblewska et al., 2017). Almajalid et al. (2018) introduced a technique based on U-Net structure, which involves an expansive path and a contracting path. The contracting path consists of convolution layers which are then followed by max pooling for downsampling, and the ReLU activation function is applied. The expansion path includes upsampling, convolution layers, and ReLU. The input images were preprocessed using speckle reduction and contrast enhancement and then post-processed to remove the noise from the segmented images.

Cho et al. (2022) introduced a multistage approach with U-Net-based residual feature selection for segmentation, followed by a classification network. The method obtained a pixel accuracy of 96.975, intersection over union (IOU) of 73.904, and DC of 82.005 on the BUSI dataset. Vakanski et al. (2020) incorporated attention blocks into a U-Net framework, enabling the model to acquire feature representations that prioritize spatial locations with notable saliency. Tang et al. (2023) presented a fully convolutional model in which the encoder output is fed to the ConvMixer model for extracting global context information. The decoder employs multiscale attention gates to enhance salient features. On the BUSI dataset, the method obtained 73.27% IOU, precision of 84.81%, F1 score of 84.16%, recall of 84.26%, and accuracy of 97.33%. Huang et al. (2021) introduced a fuzzy-based deep learning network in which breast ultrasound images are transferred to the fuzzy domain using fuzzy membership functions, which, after decreasing the uncertainty, are fed to the initial convolutional layer. The feature maps that are obtained are also converted into the fuzzy domain. The segmented results obtained are further enhanced using conditional random fields. Data augmentation is performed using a wavelet transform. Ilesanmi et al. 92021) introduced a U-Net-based method with four decoding and four encoding blocks. The method employed variant-enhanced blocks for encoding, which comprised a combined average and max pooling technique together with batch normalization. The decoding architecture utilizes double concatenated convolutions.

Tong et al. (2021) replaced the convolution module of the attention U-Net framework's networking path with the residual modules, thereby alleviating the gradient explosion problem. AbdElhakem and Torki (2023) proposed an encoder–decoder model with the ConvMixer block as the bottleneck between the encoder and decoder. Shareef et al. (2022) introduced an enhanced tumor network with a dual encoder architecture for extracting and combining image context details at various scales. It achieves this by creating feature maps using multiple kernels. These kernels extract multiscale tumor context information while conserving tumor location information. He et al. (2023) proposed a network which combines global contextual information learnt using transformer encoder blocks with convolutional neural networks for extracting features of varying resolutions. The decoder incorporates a spatial-wise cross-attention module to reduce the semantic mismatch within the encoder. The model is evaluated on three datasets: BUSI, BUS (Ilesanmi et al., 2021), and Dataset B (Huang et al., 2020).

Zhang et al. (2024) proposed a hybrid model for breast ultrasound image segmentation utilizing the long-range dependencies of transformers and the detailed local representations of convolutional neural networks. An L-G transformer block was embedded within the skip connections of the U-shaped architecture network to integrate global contextual information. The segmentation performance was enhanced by incorporating a cross-attention block module on the decoder side to facilitate interaction among different layers. The model obtained a Dice coefficient of 88.73% for the UDIAT dataset, 89.48% for the Breast Lesion Ultrasound Image dataset (BLUI) dataset (Abbasian Ardakani et al., 2023), and 83.11% for the BUSI dataset.

Zhai et al. (2022) proposed an asymmetric semi-supervised generative adversarial network, which employs a discriminator and two generators for adversarial learning. Unlabeled cases can be utilized to enhance model training as the two generators mutually guide each other to generate segmentation-predicted masks without labels. The method was evaluated on three datasets, namely DBUI, SPDBUI, ADBUI, and SDBUI.

Lin et al. (2023) proposed a dual-stage framework for the segmentation of breast lesions, utilizing transformer and Multilayer perceptron. The segmentation performance is enhanced by combining Swin Transformer block with pyramid-squeezed attention block in a parallel configuration and introducing bidirectional interactions across branches. The performance of the model is evaluated using three public datasets, namely BUSI, MT_BUS, and BUL. The BUL dataset consists of 163 images collected from the UDIAT Diagnostic Center of the Parc Taul Corporation. MT_BUS consists of 400 breast ultrasound images, with 200 images of benign breast cancer and 200 of malignant breast cancer. Table 1 compares various breast ultrasound segmentation methods.

**Table 1:** Comparison of various breast ultrasound image segmentation methods.

**References**	**Method used**	**Dataset**	**Performance measures**
Üzen (2024)	ConvMixer-based encoder and classification-based decoder	BUSI dataset with 780 images	Jaccard score–69.23% Dice score–80.23%
Zhang et al. (2023)	Model with U-Net structured segmentation branch and classification branch	1,600 breast ultrasound images	Area under curve (AUC)–99.1% Dice similarity coefficient (DSC)–89.8% Jaccard index–79.1% True positive rate (TPR)–85.9% False positive rate (FPR)–9.7%
Xu et al. (2023)	Regional attentive multitask learning framework	UDIAT dataset with 163 breast ultrasound images	Sensitivity–89.51% Specificity–99.25% DSC–85.69% Accuracy–98.79% Intersection over union (IOU)–77.84%
		BUSI dataset with 780 images	Sensitivity–82.54% Specificity–98.00% DSC–80.04% Accuracy–96.4% IOU–71.93%
Chen et al. (2023)	Squeeze-and-excitation attention U-Net	BUSI dataset with 780 images	Jaccard–70.36% Precision–79.73% Recall–82.70% Specificity–97.42% Dice–78.51%
		Dataset B with 163 breast ultrasound images	Jaccard–73.17% Precision–82.58% Recall–84.02% Specificity–99.05% Dice–81.50%
Lyu et al. (2023)	Enhanced Pyramid Attention Network integrating multi-scale features and attention mechanism	BUSI dataset 780 images	Accuracy–97.13% DSC–80.71% IOU–68.53% Recall–79.30% Precision–83.50% Specificity–98.54%
		OASBUD dataset (Piotrzkowska-Wróblewska et al., 2017) with ultrasound scans	Accuracy–97.97% DSC–79.62% IOU–67.52% Recall–74.43% Precision–87.92% Specificity–99.38%
Almajalid et al. (2018)	U-Net architecture	221 breast ultrasound images	DSC–82.52% SI–69.76% False negative–21.34% FPR–18.59% TPR–78.66%
Cho et al. (2022)	Multistage segmentation method with classification and segmentation networks	BUSI dataset with 780 breast ultrasound images	Accuracy–97.253% IOU–77.835% DSC–84.856%
		UDIAT dataset with 163 breast ultrasound images	Accuracy–98.601% IOU–77.094% DSC–85.366%
Vakanski et al. (2020)	U-Net architecture with attention blocks	Dataset of 510 breast ultrasound images collected from three different hospitals.	DSC–90.5% Jaccard index–83.8% TPR–91.0% FPR–8.9% Accuracy–98% AUC–ROC–95.7%
Tang et al. (2023)	Encoder–decoder structure with ConvMixer bottleneck and multiscale attention gates	BUSI with 780 breast ultrasound images	IOU–73.27% Recall–84.26% Precision–84.81% F1-value–84.16% Accuracy–97.33%
Huang et al. (2021)	Fuzzy, fully convolutional neural network	Dataset with 325 breast ultrasound images	TPR–90.33% FPR–9.00% IOU–81.29%
Ilesanmi et al. (2021)	VEU-Net	Dataset with 264 images	Hausdroff distance–7.81 Jaccard measure–80.07% Dice measure–90.82%
		Dataset with 830 images	Hausdroff distance–7.71% Jaccard measure–79.49% Dice measure–90.67%
Tong et al. (2021)	U-Net with extended residual convolution and residual convolution	Dataset of 316 breast ultrasound images	Dice–92.8% Specificity–97.9% Sensitivity–85.0% Accuracy–95.9% AUC–94.1% F1 score–87.3% Recall–84.6% Precision–90.2%
AbdElhakem and Torki (2023)	Encoder–decoder structure with ConvMixer block as bottleneck	BUSI dataset with 780 breast ultrasound images	IOU–68.17% Dice score–80.60%
Shareef et al. (2022)	Enhanced small tumor-aware network	BUSI dataset with 780 breast ultrasound images	TPR–80% FPR–36% Jaccard Index–70% DSC–78%
		BUSIS dataset with 562 images	TPR–91% FPR–7% Jaccard index –86% DSC–92%
		Dataset B with 163 BUS images	TPR–84% FPR–22% Jaccard index –74% DSC–82%
He et al. (2023)	Hybrid CNN transformer with transformer encoder blocks and spatial-wise cross-attention in the decoder.	BUSI dataset with 780 breast ultrasound images	Dice–82% Accuracy–96.94% Jaccard–71.84% Recall–82.14% Precision–83.24% HD–34.55%
		BUS dataset with 163 breast ultrasound image dataset	Dice- 84.13% Accuracy–98.49% Jaccard–73.83% Recall–83.19% Precision–88.50% Hausdorff distance–21.66%
		Dataset B with 320 images	Dice–97.23% Accuracy–97.41% Jaccard–94.63% Recall–97.33% Precision–97.14% Hausdorff distance–19.35%
Zhang et al. (2024)	Hybrid CNN transformer with L-G transformer block is embedded into the skip connections of the Ushape architecture and cross-attention module on the decoder.	UDIAT dataset	Dice coefficient–88.73 ± 2.11 Hausdorff distance–3.64 ± 2.26 IOU–81.22 ± 2.30 Accuracy–99.03 ± 0.32 Specificity–99.60 ± 0.12 Precision–88.68 ± 2.25
		BLUI dataset	Dice coefficient–89.48 ± 0.44 Hausdorff distance–5.38 ± 0.66 IOU–82.12 ± 0.85 Accuracy–96.96 ±0.42 Specificity–98.17 ± 0.29 Precision–89.93 ± 1.15
		BUSI dataset	Dice coefficient–83.11 ± 2.07 Hausdorff distance–10.67 ± 2.44 IOU–75.26 ± 2.08 Accuracy–96.80 ±0.16 Specificity–98.52 ± 0.24 Precision–86.08 ± 2.52
Zhai et al. (2022)	Asymmetric semi-supervised generative adversarial network	DBUI	IOU–0.7683 Accuracy–0.9760 Dice coefficient–0.8690
		SPDBUI	IOU–0.8852 Accuracy–0.9508 Dice coefficient–0.9391
		ADBUI	IOU–0.6187 Accuracy–0.9605 Dice coefficient–0.7644
		SDBUI	IOU–0.7123 Accuracy–0.9589 Dice coefficient–0.8319
Lin et al. (2023)	Transformer and multilayer perceptron	BUSI	Dice: Benign–0.8127 ± 0.2178, malignant–0.6939 ± 0.2401 IOU: Benign–0.7269 ± 0.2370, malignant–0.5754 ± 0.2448 Precision: Benign–0.7932 ± 0.2382, malignant–0.6943 ± 0.2594 Sensitivity: Benign–0.8873 ± 0.1950, malignant–0.7679 ± 0.2588 HD: Benign–3.75 ± 1.83, malignant–5.88 ± 1.61
		MT_BUS	Dice–0.8016 ± 0.1722 IOU–0.6975 ± 0.2030 Precision–0.8021 ± 0.1976 Sensitivity–0.8465 ± 0.1780 HD–4.72 ± 2.04
		BUL	Dice–0.8698 ± 0.1200 IOU–0.7852 ± 0.1502 Precision–0.8938 ± 0.1263 Sensitivity–0.8717 ± 0.1374 HD–3.30 ± 1.18

## 3 Proposed methodology

**Figure 1:**
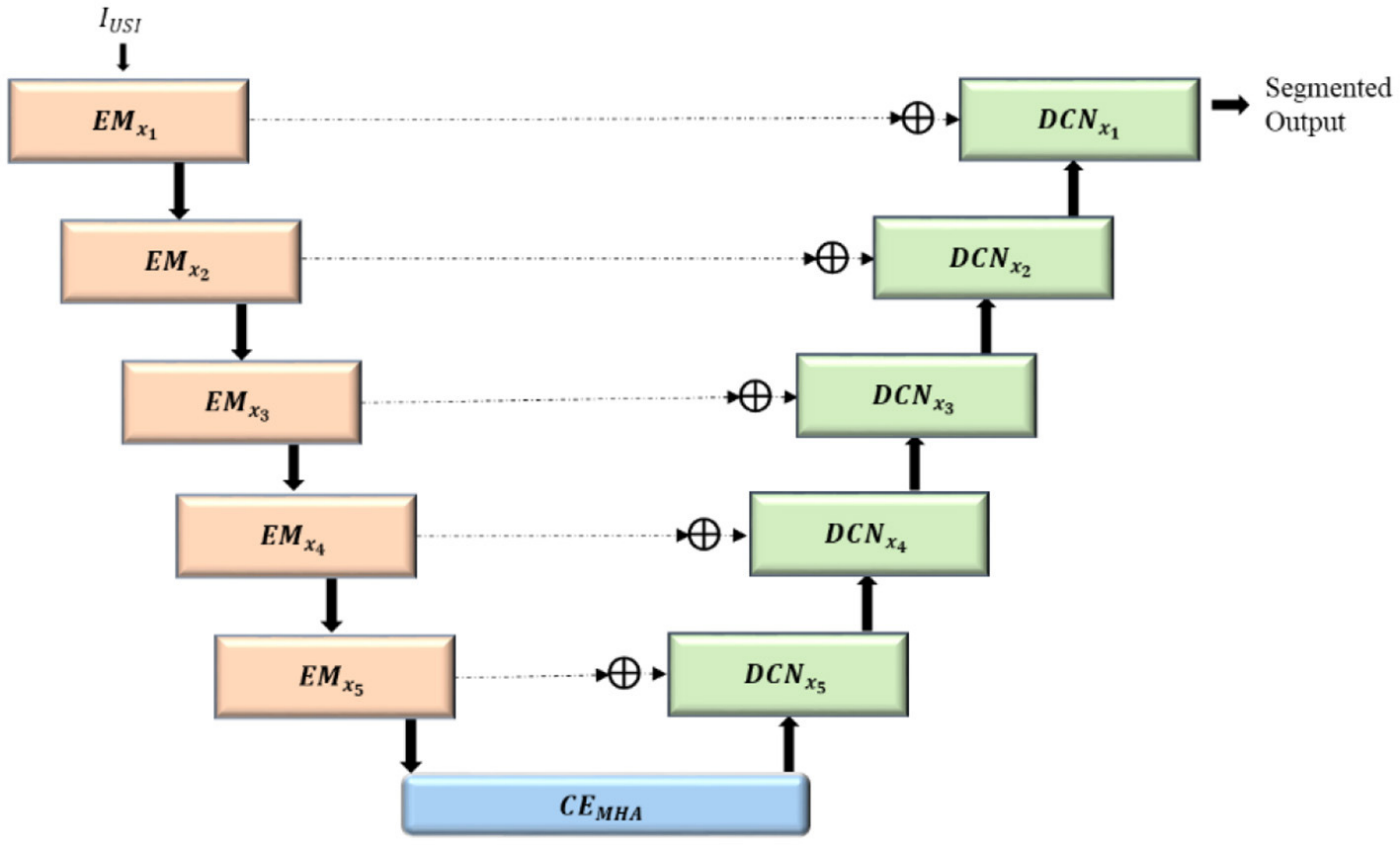
Architecture of the HMA-net.

The proposed hybrid mixer framework with multihead attention (HMA-Net) incorporates a lightweight spatial-channel mixing model within its encoder (*EM_x__1_* to *EM_x__5_*) to extract robust features effectively. convolution-enhanced multihead attention module (*CE*_*MHA*_) serves as the bottleneck between encoder and decoder, enhancing the long-range dependencies and allowing it to focus on subtle differences for precise segmentation. In the decoder (*DCN_x__5_* to *DCN_x__1_*), enhanced ConvNeXT (ECN) modules facilitate upsampling and high-resolution reconstruction, refining features and accurately capturing boundaries and contours in breast ultrasound images. Figure 1 displays the architecture of the hybrid mixer framework with multihead attention (HMA-Net).

### 3.1 ConvMixer-based encoder blocks (**EM**_**x**_**i**__**)**

**Figure 2:**
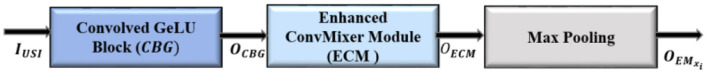
ConvMixer-based encoder block.

The complex features from the input ultrasound images (*I*_*USI*_) are extracted using five consecutive encoder mixer blocks *EM*_*x*_1__ to *EM*_*x*_5__. Downsampled feature maps with reduced dimensions (*O*_*E*_*M*__*x*_*i*___) are generated, facilitating an enhanced hierarchical representation of complex features. The enhanced ConvMixer modules (*ECMs*) incorporated squeeze and excitation along with residual linking for modeling channelwise interdependencies by adaptively adjusting channel feature responses. The structure of ConvMixer-based encoder block is shown in Figure 2.

#### 3.1.1 Convolved GeLU block (**CBG**)

*CBG* block is designed to capture edge information for accurate boundary identification. The batch normalization component stabilizes training and accelerates convergence, while the Gaussian Error Linear Unit (GeLU) activation introduces enhanced non-linearity, enriching the extracted features. Convolutions with filters of size 3 × 3 are performed to generate feature maps emphasizing distinct features of the input image by extracting local features. The generated feature maps are stabilized and normalized by batch normalization (*BN*), accelerating faster convergence and enhancing the resilience of the model to variations in the input data distribution. Non-linearity is introduced by the GeLU activation function (γ), enhancing the ability of the model to acquire intricate relationships within the data and to make accurate predictions on unfamiliar data. The functioning of the Convolved GeLU block (CBG) is shown in Equation 1:


(1) $$\begin{array}{l} O_{CBG}=\gamma \left(BN\left(C_{3\times 3}\left(I_{USI}\right)\right)\right) \end{array}$$

#### 3.1.2 Enhanced ConvMixer module

Enhanced ConvMixer module (ECM) integrate depthwise convolutions, pointwise convolutions and squeeze and excitation to improve feature extraction and boost the representation power of the model. Local patterns in the input images are detected using depthwise convolutions (*D*_3 × 3_), where each input channel is convolved *via* separate convolutions instead of applying the same kernel to all channels, thus extracting spatial features while maintaining channel independence. Non-linearity is introduced by passing the feature maps through the GeLU activation function, allowing the model to learn complex patterns with improved gradient flow, thus aiding the model to learn slight variations in input features. Training is accelerated and stabilized by normalizing the non-linearly transformed feature map.

Channel-wise features are generated by pointwise convolutions (*P*_1 × 1_), by mixing the information across the channels, with the application of a 1 × 1 convolution filter to each and every pixel across all the channels. The spatial and channel features are integrated by *CBG* block, facilitating improved integration of information across channels, thereby boosting the network's ability to represent complex patterns. Spatial features captured by depthwise convolutions and channel features captured by 1 × 1 convolutions are enhanced by the GeLU activation function, enabling the network to learn complex patterns. The output of the convolutions is normalized using batch normalization to ensure that the activations have a reliable and consistent distribution (*O*_*IECM*_), as shown in Equation 2.


(2) $$O_{IECM}=\;BN(\gamma (P_{1\times 1}\left(O_{CBG}\left(BN\left(\gamma \left(D_{3\times 3}\left(O_{CBG}\right)\right)))\right)\right)\right)$$

The attention mechanism is incorporated into the *ECM* module by adding squeeze-and-excitation (*S*_*E*_) block, thereby enhancing the model accuracy by giving higher priority to significant features and reducing the impact of less useful ones. It is a process of adaptively adjusting the weights of each feature map to selectively enhance the weight of relevant feature maps, which in turn improves the representation power of the model. Global average pooling (*G*_*AP*_) is applied to the feature maps thereby generating a single value for each channel, hence reducing the spatial dimensions. The global average pooled vector is mapped into a low-dimensional space using a dense layer, and the network representation capacity is enhanced by introducing non-linearity using the ReLU activation function (*D*_*RL*_). The reduced dimensional vector is mapped back to its original size using a fully connected layer, and the feature maps are scaled with sigmoid activation functions (*D*_*SD*_) to generate channel-wise weights. The channel-wise weights generated are reshaped (*R*_*s*_) to match with the dimensions of the input feature map, as given in Equation 3.


(3) $$O_{SE}=(R_{s}(D_{SD}(D_{RL}(G_{AP}(O_{IECM})))))$$

The *S*_*E*_ block's output (_*O*_*S*_*E*_), that is, the channel-wise weights generated are multiplied with its input feature map in order to recalibrate the feature maps, as shown in Equation 4.


(4) $$\begin{array}{l} O_{SE}={O_{S}}_{E}⊗O_{IECM} \end{array}$$

Ultimately, the *ECM* reintegrates the recalibrated feature maps with the input using a residual link, which facilitates the flow of gradients and improves the network's ability to represent information, as in Equation 5.


(5) $$\begin{array}{l} {O_{ECM}=O}_{SE}⊕O_{CBG} \end{array}$$

**Figure 3:**
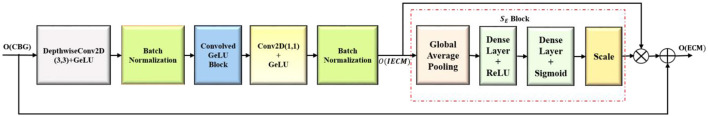
Enhanced ConvMixer module (**ECM**).

The architecture of the ECM is given in Figure 3.

#### 3.1.3 Max pooling

The feature maps from the *ECM* blocks are downsampled (*M*_2*X*2_), as in Equation 6, to enhance the learning ability of the model by capturing high-level features at varying spatial scales. Translational invariance is provided so that the model can detect lesions irrespective of their position in the image, which results in improved generalization.


(6) $$\begin{array}{l} O_{EM_{x_{i}}}=M_{2X2}\left(O_{ECM}\right) \end{array}$$

### 3.2 Convolution-enhanced multihead attention module (**CE**_**MHA**_**)**

**Figure 4:**
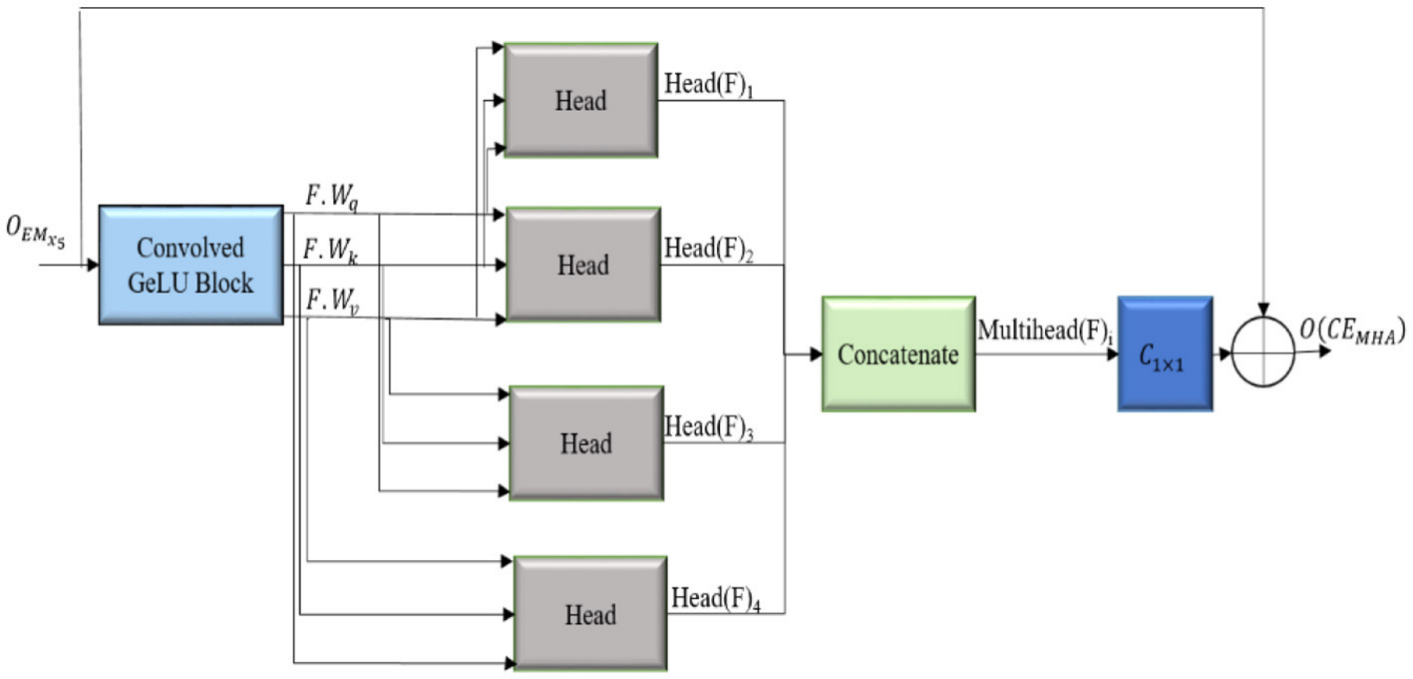
Architecture of the convolution enhanced multihead attention (**CE**_**MHA**_) module.

The convolution-enhanced multihead attention (*CE*_*MHA*_) emphasize the relevant features across distinct regions of the image, ensuring that the masks generated by the decoder will closely follow the lesion boundaries, thus aiding in the accurate identification of lesions. The architecture of the *CE*_*MHA*_ module is shown in Figure 4.

The downsampled feature maps from the *EM*_*x*_*i*__ blocks are enhanced by the convolved GeLU block, thus improving the ability of the model to process and comprehend the underlying structure of the input data and stabilizes the training process. Long-range diverse dependencies across different parts of the images are captured using four distinct heads, each of which focuses on a specific pattern in the image, thus allowing the model to maintain context by comprehending the relation between different areas of the image. The enhanced feature map F is linearly transformed into a query(*F*.*W*_*q*_), key(*F*. *W*_*k*_) and value (*F*.*W*_*v*_) matrices, which, in turn, calculate the attention score. The similarity between the query and key matrices is calculated $\left((F.W_{q}){\left(F.W_{k}\right)}^{T}\right)$ and scaled by $\left(\sqrt{\frac{d}{h}}\right)$ to stabilize for larger dimensions. The attention scores are normalized using the softmax activation function (σ), and the most significant features from the input feature map are aggregated, allowing the model to integrate global and local contextual details. The computations inside each attention unit (h) are shown in Equation 7. The output dimension of each attention head is $\frac{d_{model}}{h}$ where *d*_*model*_ represents the dimensionality of the model.


(7) $$Head{\left(F\right)}_{i}=F.W_{v\;}\sigma \left[\frac{\left(F.W_{q}\right){\left(F.W_{k}\right)}^{T}}{\sqrt{\frac{d}{h}}}\right]\in {\mathbb{R}}^{\frac{d_{model}}{h}\times n}$$

The attention scores calculated by the four distinct heads are merged to obtain *Multihead*(*F*)_*i*_ , as in Equation 8, with output dimension *d*_*model*_, facilitating the ability of the model to concentrate on distinct segments of the input image concurrently, effectively collecting many facets of the data. The model is thus enabled to generalize better for different types of input images.


(8) $$Multihead{\left(F\right)}_{i}=Con\left[head{\left(F\right)}_{1},\ldots ,head{\left(F\right)}_{h}\right]\in R^{d\times n}$$

The output of the attention is transformed back to its original number of channels by convolving with a filter of size 1 × 1 (*C*_1 × 1_). The refined feature maps generated are appended with its input to enable the smooth flow of gradients throughout the network, thereby alleviating the issues of vanishing gradient problems and aiding in learning efficient representations, as shown in Equation 9.


(9) $$\begin{array}{l} O\left(CE_{MHA}\right)=\left(\left(C_{1\times 1}\left(Multihead\left(O_{CBG}\left(F\right)\right)\right)\right)⊕F\right) \end{array}$$

The residual connections are integrated to enhance segmentation accuracy and training stability Equation 9. The actual input is integrated with the attention-enhanced features, allowing the model to combine both the original features and globally attended information. This approach promotes the smooth flow of gradients and reduces the vanishing gradient problem. Intricate contextual information is preserved, facilitating the precise delineation of tumor boundaries. The convolution-enhanced multihead attention with residual connections (*CE*_*MHA*_) ensures the balanced integration of learned attention-driven features while maintaining training stability and efficient convergence.

### 3.3 ConvNeXT-based decoder blocks (**DCN**_**x**_**i**__)

**Figure 5:**
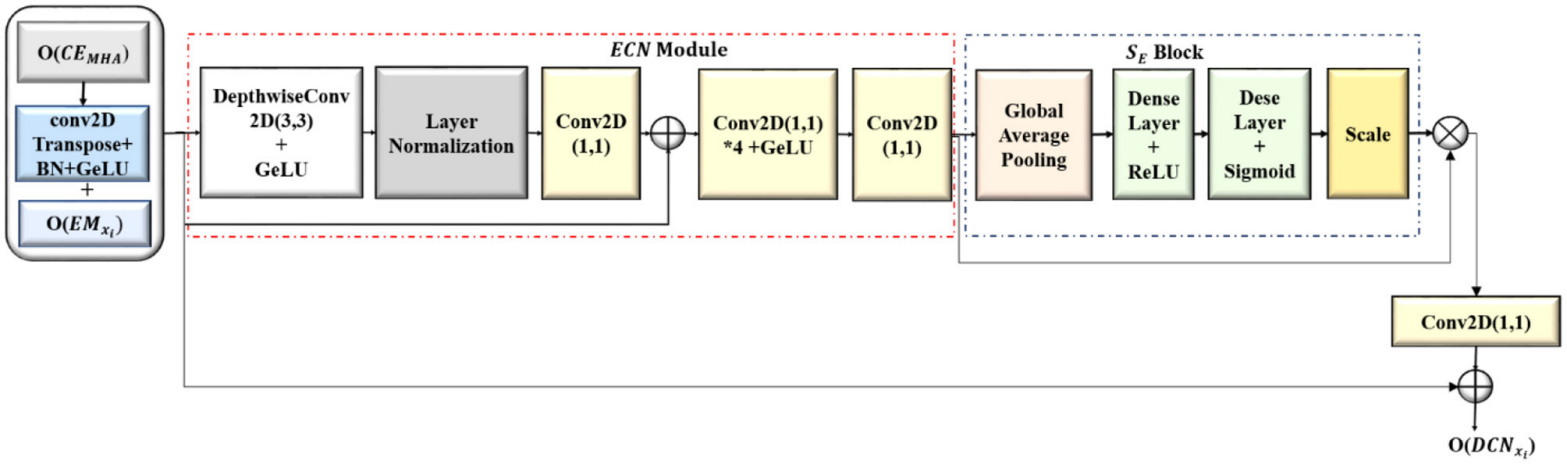
Architecture of the ConvNeXT-based decoder (**DCN**_**x**_**i**__**)** block.

The output feature maps from the convolution-enhanced multihead attention (*O*(*CE*_*MHA*_)) are upsampled and concatenated with the corresponding feature maps from the *EM*_*x*_*i*__, which increased the resolution of the feature maps to that of the original image, and the segmentation masks were generated. The detailed architecture of the ConvNeXT-based decoder blocks (*DCN*_*x*_*i*__) are shown in Figure 5. In each *DCN*_*x*_*i*__ block with enhanced ConvNeXT (*ECN*), the spatial dimensions of the downsampled refined feature map are increased by transposed convolutions (*C*_*T*_2 × 2__) while lowering the number of channels. The upsampled feature map from the transposed convolutions is batch normalized to accelerate the training process, ensuring that the input to the succeeding GeLU activation layer has a uniform distribution.

The downsampled feature maps from each *EM*_*x*_*i*__ block after center cropping (*Cr*(_*O*_*E*_*M*_*xi*__) ) has been concatenated with the corresponding upsampled feature map from *DCN*_*x*_*i*__ block, resulting in a merged feature map by combining semantic information with the spatial information. This enabled the *DCN*_*x*_*i*__ to get a comprehensive understanding of the data, utilizing both the low level and high level features. The concatenated feature map is further refined with the Enhanced ConvNeXT (*ECN*) module as shown in Equation 10.


(10) $$O_{DCN_{xi}}=(f_{ECN}(\gamma (BN(C_{T_{2\times 2}}(O(CE_{MHA})))))+Cr(O_{EM_{xi}}))$$

Enhanced ConvNeXT module extracts spatial features independently from each channel by convolving separately with filters of size 7 ((*D*_*conv*_(*x*)) resulting in feature maps with better representation. The spatially significant features are normalized to mitigate the effect of internal covariance shift during training by calculating the mean and variance of the inputs of every individual sample (*L*_*N*_). The normalized features are mapped back to the original dimensions (*C*_1 × 1_) and the input (x) is added. The mixing of features across the channels is enhanced by two 1 × 1 convolutions, with the first convolution expanding the feature channels by 4 ($C_{1\times 1}^{4*}$) which enhanced the feature refining and mixing capacity of the model before bringing it back to its original number of filter channels with the second convolution(*C*_1 × 1_). The capacity of the model to recalibrate channel-specific feature responses is improved by integrating squeeze-and-excitation (*S*_*E*_) block, highlighting informative features and reducing the prominence of less valuable ones, which results in enhanced segmentation accuracy and improved feature refinement while generating the image segments. The functioning of the ECN module is given in Equation 11.


(11) $$f_{ECN}=S_{E}(C_{1\times 1}(\gamma (C_{1\times 1}^{4^{*}}(C_{1\times 1}(L_{N}(D_{conv}(x)))+x))))$$

### 3.4 Combined loss function

A combined loss function, which integrates two loss functions, is used for training the model, by which the segmentation performance can be optimized. The discrepancy between the actual label and the predicted label is measured by using the binary cross entropy function and performs best when the data distribution is uniform. However, this alone cannot be used for training where the tumor occupies only a small fraction of the image due to class imbalance.

To address this issue, dice loss is integrated with binary cross entropy. The extent to which the true mask and predicted mask overlapped is assessed using dice loss function, with an emphasis on the regions where the two intersect. The combined loss is calculated as in Equation 12.


(12) $$\begin{array}{l} Combined\;loss=\frac{1}{M}\sum_{i=1}^{N}-\left[y_{i}.\log\left({\bar{y}}_{i}\right)+\log\left(1-{\bar{y}}_{i}\right).\left(1-y_{i}\right)\right]\;\\ \;+1-\frac{2.\sum_{i=1}^{N}y_{i}.{\bar{y}}_{i}}{\sum_{i=1}^{N}\sum_{i=1}^{N}{\bar{y}}_{i}} \end{array}$$

Here, ${\bar{y}}_{i}$ is the predicted probability, M is the total number of samples, and *y*_*i*_ is the actual label. Binary cross entropy loss guarantees that each pixel is classified correctly and, the accurate segmentation is ensured by dice loss. The performance of the model is enhanced by the effective utilization of these two losses.

## 4 Experimental results and discussions

This section offers a detailed description of the dataset, experimental setup, data preprocessing and augmentation methods, evaluation metrics, ablation study, and performance evaluation. Various performance measures are utilized to access the performance of the proposed HMA-Net.

### 4.1 Dataset description

**Figure 6:**
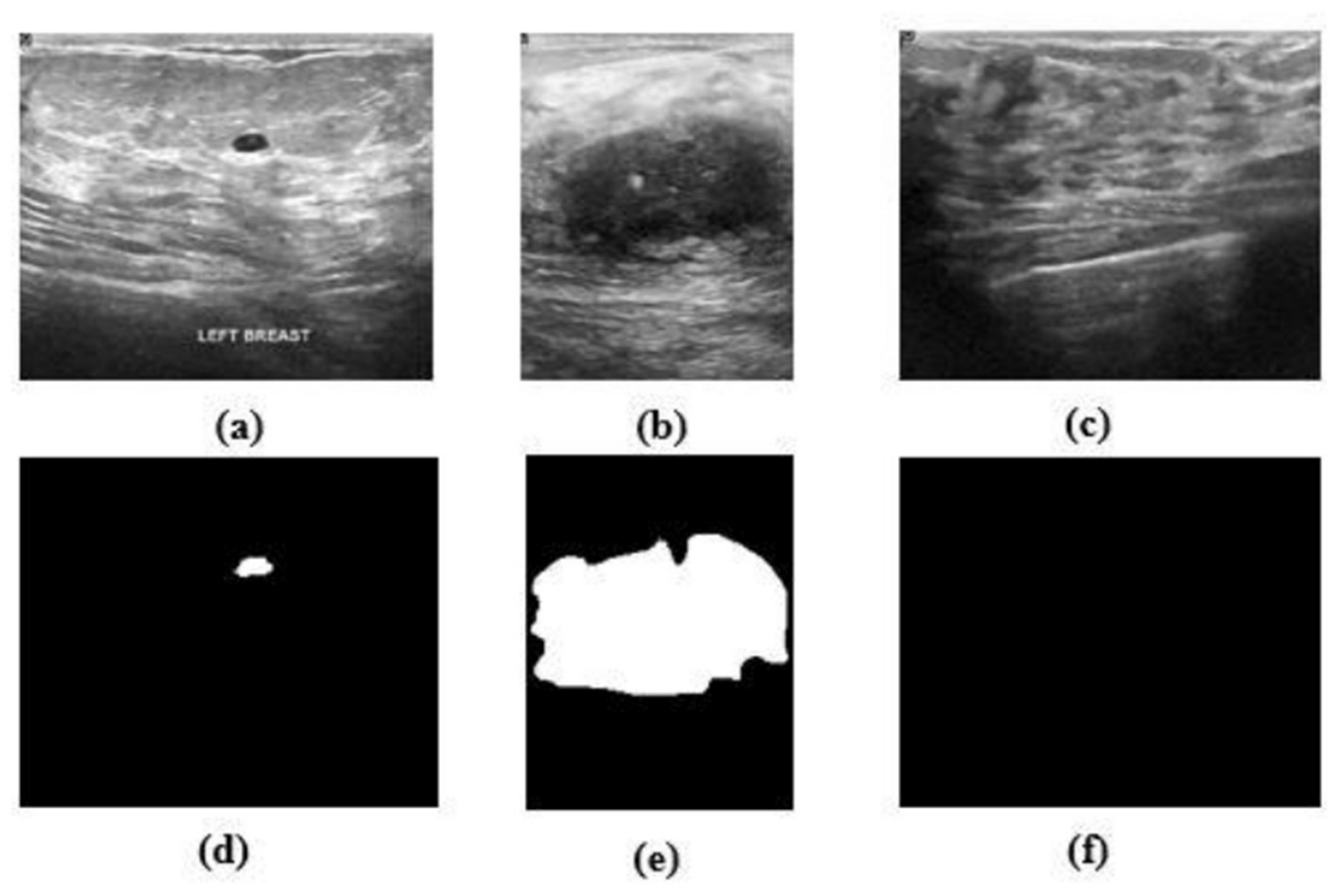
**(a–c)** Benign, malignant, and normal images from the BUSI dataset. **(d–f)** Masks corresponding to **(a–c)**.

The proposed *HMA Net* model is validated on two datasets—the BUSI dataset and the BrEaST dataset. The first dataset used is BUSI, which is a public benchmark dataset with 780 PNG images categorized into three classes—benign, malignant, and normal. Each image of size 500 × 500 pixels is further enhanced by a corresponding ground truth annotation that offers precise segmentation masks for the tumors. The data were gathered from a group of 600 female patients, ranging in age from 25 to 75, during the year 2018 at the Baheya Hospital. Sample images and masks from the BUSI dataset are shown in Figure 6. The BUSI dataset presents a balanced depiction of various breast abnormalities through a varied assortment of benign, malignant, and normal cases of breast ultrasound images. It is ideal for segmentation model evaluation and training in practical clinical circumstances due to its diversity.

**Figure 7:**
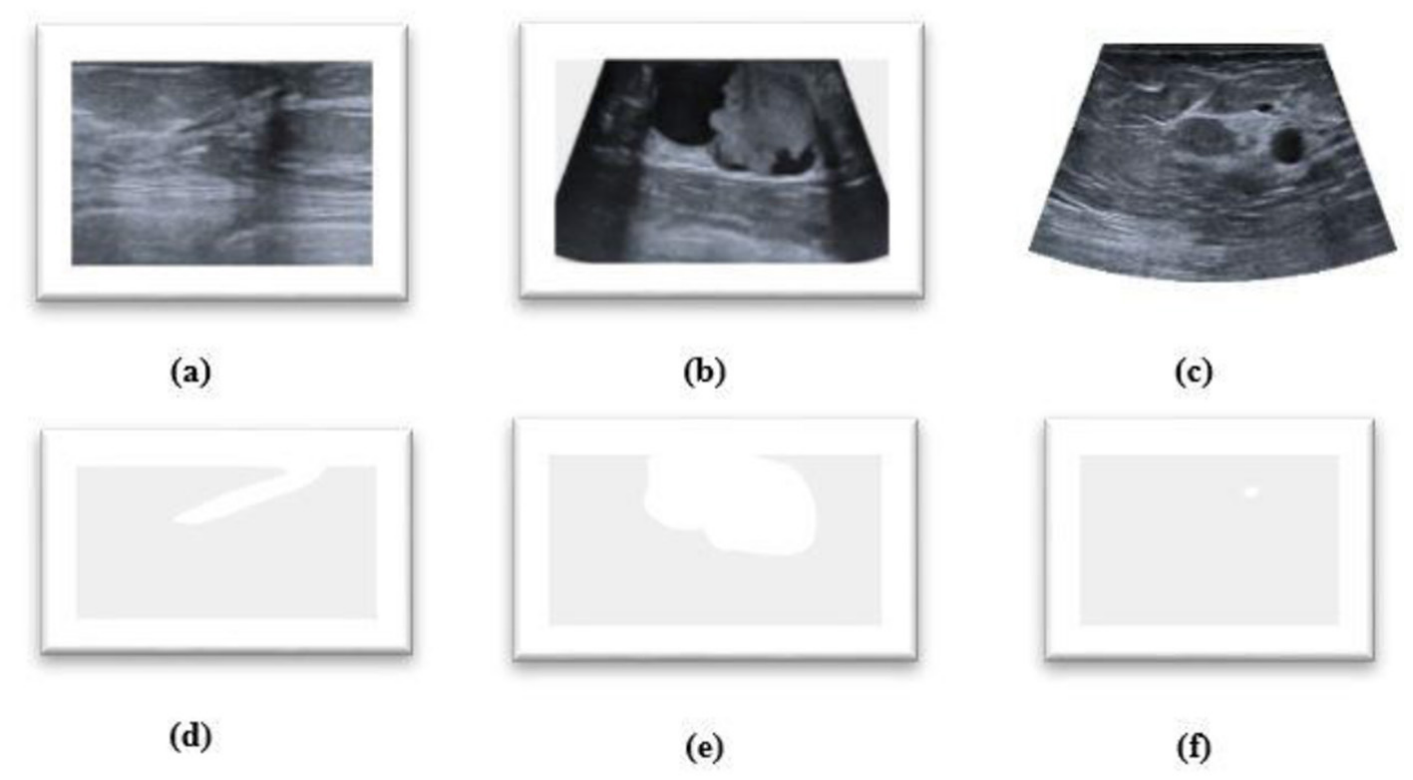
**(a–c)** Breast ultrasound images. **(d–f)** Masks from the BrEaST dataset.

The BrEaST dataset is comprised of 256 images obtained from 256 patients. The dataset comprises 98 instances of cancer, 154 instances of benign lesions, and four instances of normal tissue images. The initial stage in constructing the dataset involved anonymising, gathering, and transferring the data. In order to safeguard the confidentiality of patients, any identifiable data have been eliminated from the images. Figure 7 shows sample images and masks from the BrEaST dataset. In order to ensure high-quality and clinically appropriate labels for both tumors and surrounding areas, the BrEaST dataset was manually annotated by skilled radiologists. For accurate model evaluation in medical image segmentation tasks, this level of precision is necessary. The dataset is divided into two parts: 20% is used for testing and 80% is used for training.

### 4.2 Experimental setup

The task was implemented on a cloud computing platform known as Google Colab notebooks. The utilization of this cloud-based technology facilitated the training and execution of the deep learning model with enhanced efficiency. The *HMA* − *Net* model was implemented using the Python programming language and various important libraries, such as Keras, matplotlib, Tensorflow, OS, and sklearn, were used throughout the implementation process.

### 4.3 Data preprocessing and augmentation

**Figure 8:**
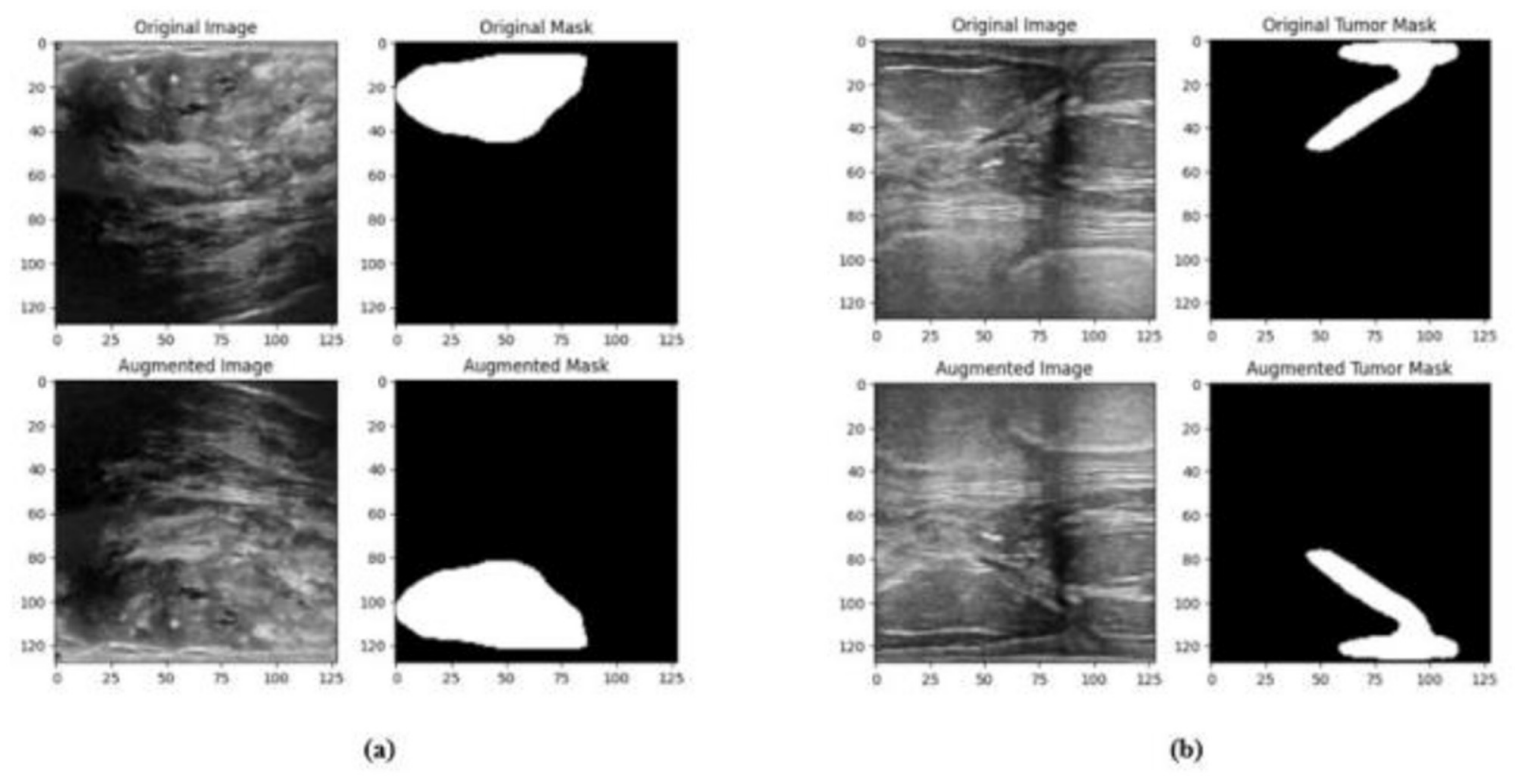
Sample augmented images and masks from the **(a)** BUSI dataset, **(b)** BrEaST dataset.

In order to improve the effectiveness of the network training process, the size of the input image of the network structure is resized to 128 × 128. A significant amount of training data is necessary for deep neural networks in order to obtain performance levels that are adequate. The process of data augmentation is carried out with the purpose of artificially increasing the quantity of the dataset by generating new versions of the images that are already there, thus overcoming the issues that are associated with having limited data. Random flips in horizontal and vertical directions are applied to the masks and images. Images and masks are randomly shifted horizontally by up to 10% of their width and randomly shifted vertically by up to 10% of their height. Images and masks are randomly zoomed up to 20% and are randomly rotated with a rotation angle of up to 20 degrees. The pixels that move outside of the image are filled by fill_mode to the nearest, which fills the empty region with the pixel that is adjacent to it. The sample augmented data from the BUSI and BrEaST datasets are displayed in Figures 8a, b, respectively.

### 4.4 Evaluation metrics of the proposed architecture

The model is accessed using various evaluation metrics, including the Jaccard similarity index (JI), accuracy, Dice similarity coefficient (DSC), precision, recall, and AUC. The Jaccard index is employed to assess the degree of similarity or variation among sets. The Jaccard index can be used to determine how similar the predicted image is to the ground truth image. Let P be the ground truth mask and Q be the predicted mask; the Jaccard index is given as in Equation 13.


(13) $$\begin{array}{l} JI\left(P,Q\right)=\frac{\sum \left(P.Q\right)}{\sum \left(P+Q-P.Q\right)} \end{array}$$

The DSC is a statistical metric employed to assess the resemblance between two sets of data. The measurement quantifies the extent to which the predicted segmentation mask and the ground truth mask overlap. The equation for DSC is given in Equation 14.


(14) $$\begin{array}{l} DSC=\frac{2|P\cap Q|}{\left|P|+|Q\right|} \end{array}$$

Segmentation accuracy is the metric used to evaluate the pixels that are correctly classified in the image that has been segmented. It is defined as the ratio of the total number of true positive and true negative pixels to the total number of pixels in the image, as in Equation 15.


(15) $$\begin{array}{l} Accuracy=\frac{TP+TN}{TP+TN+FP+FN} \end{array}$$

Recall measures the ability of the model to accurately detect tumor areas. It is the percentage of actual positive cases (tumor pixels) that the segmentation model correctly identifies as positive, as given by Equation 16.


(16) $$\begin{array}{l} Recall=\frac{TP}{TP+FN} \end{array}$$

Precision is defined as the ratio of the number of pixels that are correctly predicted as tumorous to the total number of samples that are predicted as tumorous as in Equation 17.


(17) $$\begin{array}{l} Precision=\frac{TP}{TP+FP} \end{array}$$

AUC refers to area under receiver operating characteristic curve in which true positive rate (TPR) is plotted against false positive rate (FPR). The higher the AUC, the better the performance of the model.

### 4.5 Ablation studies

**Figure 9:**
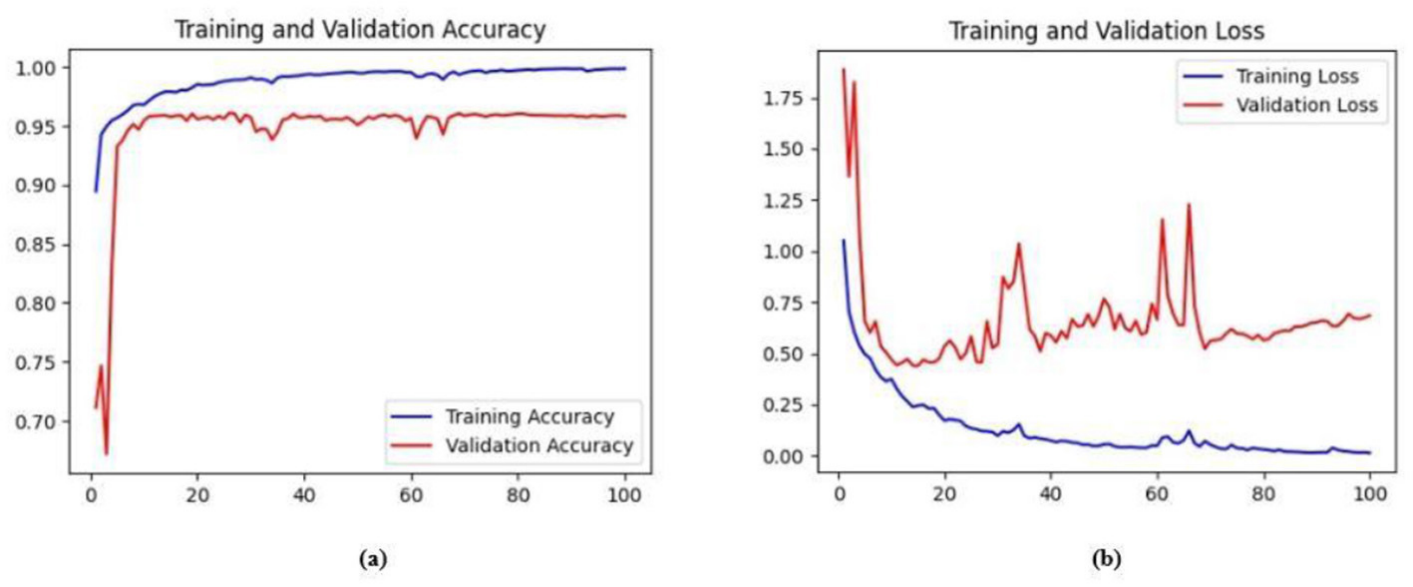
**(a)** Training and validation accuracy for Model 1 on the BUSI dataset. **(b)** Training and validation loss for Model 1 on the BUSI dataset.

**Figure 10:**
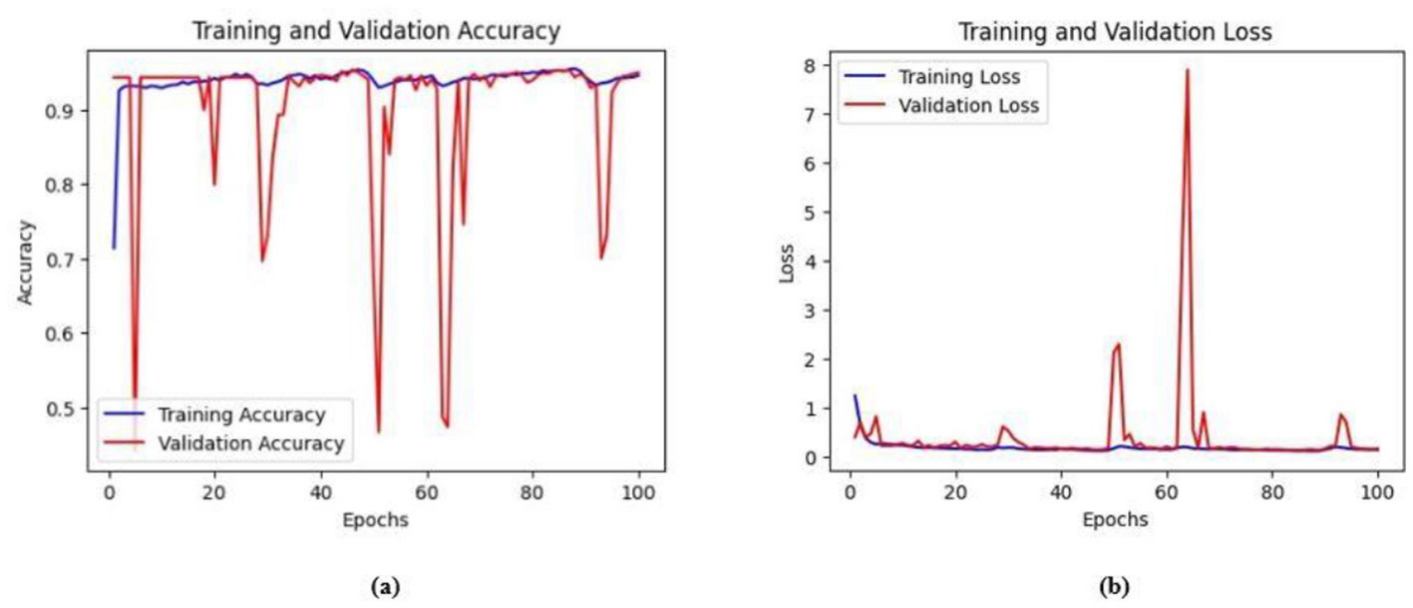
**(a)** Training and validation accuracy for Model 1 on the BrEaST dataset. **(b)** Training and validation loss for Model 1 on the BrEaST dataset.

**Figure 11:**
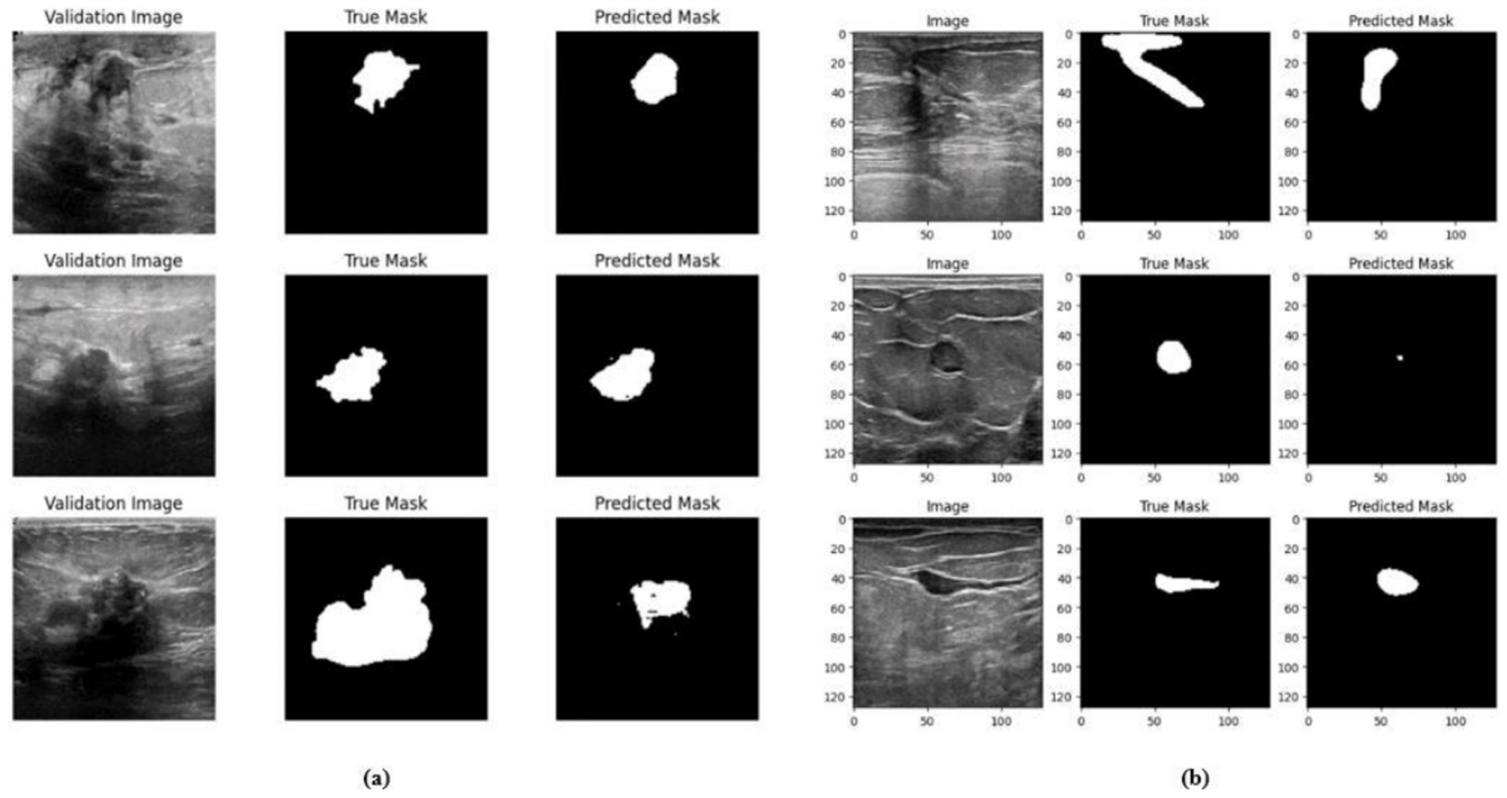
Visualizations of the segmentation result for the Model 1 on the **(a)** BUSI dataset **(b)** BrEaST dataset.

**Figure 12:**
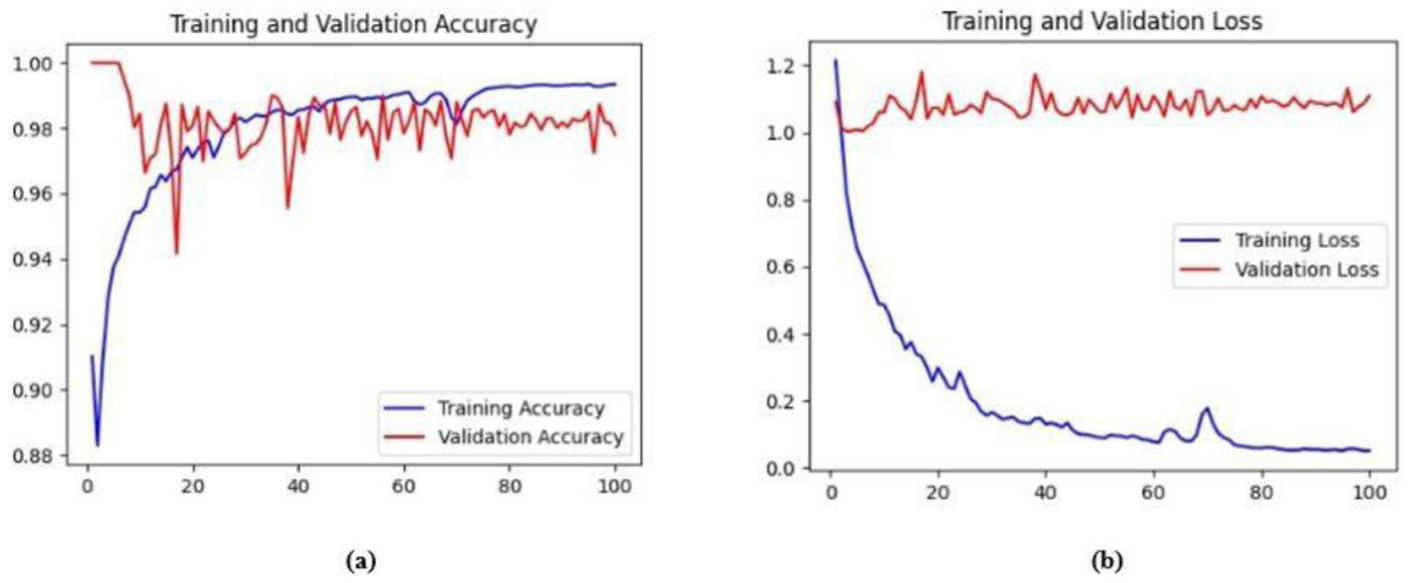
**(a)** Training and validation accuracy of Model 2 on the BUSI dataset. **(b)** Training and validation loss of Model 2 on the BUSI dataset.

**Figure 13:**
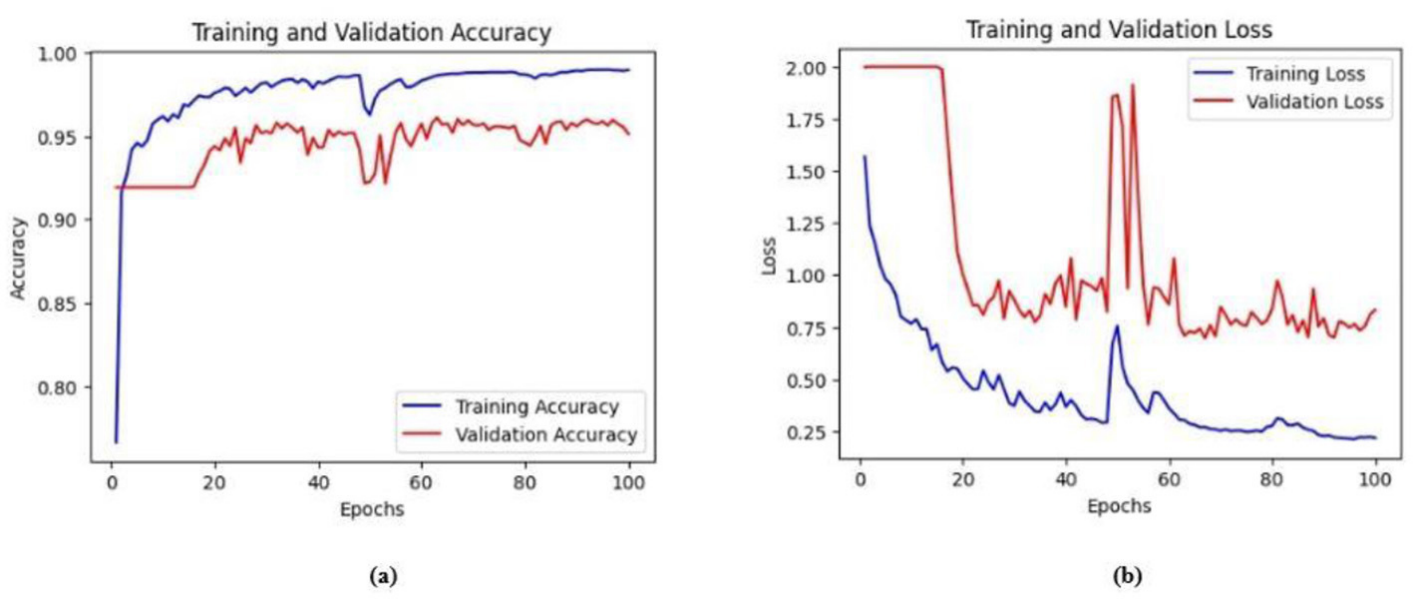
**(a)** Training and validation accuracy of Model 2 on the BrEaST dataset. **(b)** Training and validation loss of Model 2 on the BrEaST dataset.

**Figure 14:**
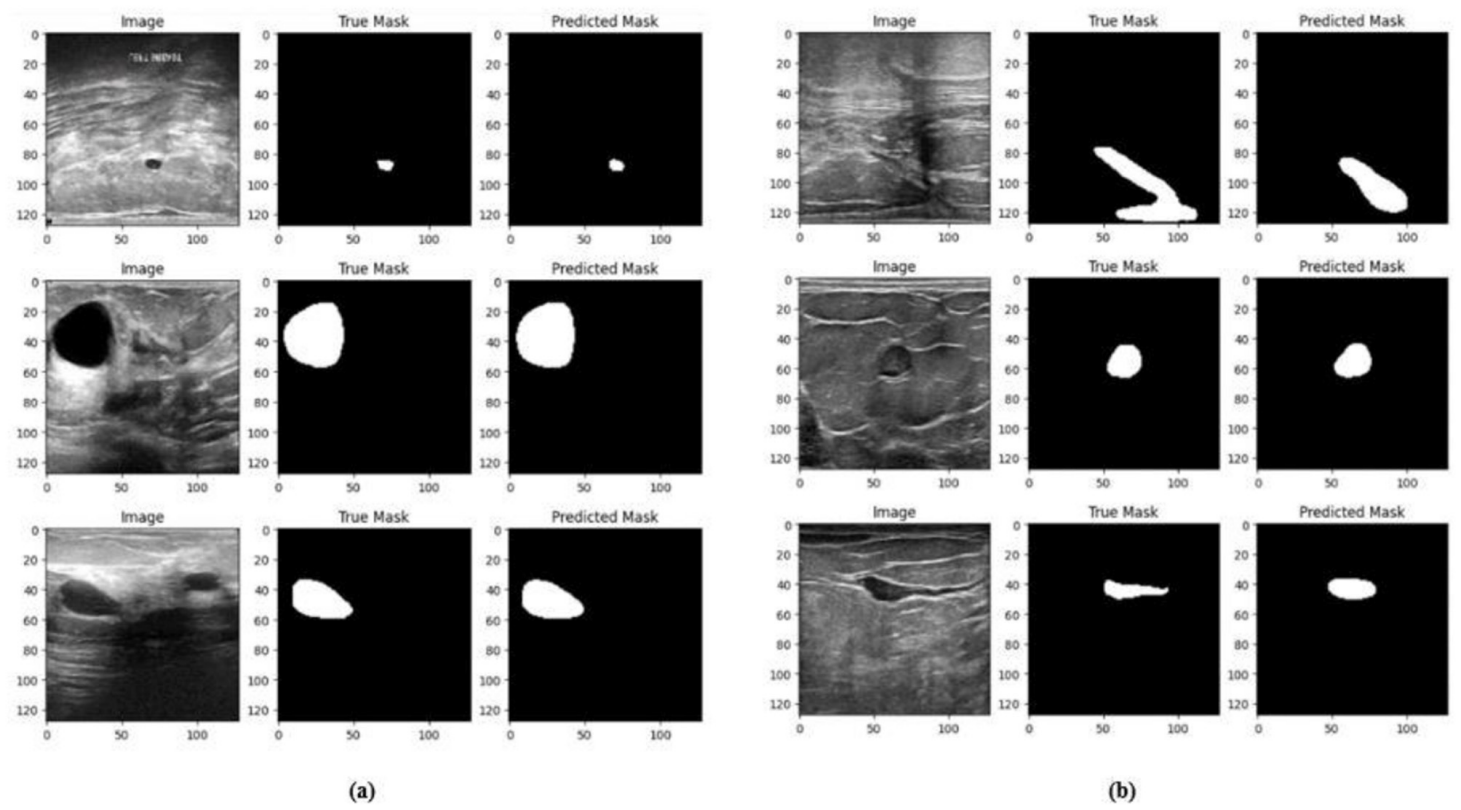
Visualizations of the segmentation results of Model 2 on the **(a)** BUSI dataset **(b)** BrEaST dataset.

**Figure 15:**
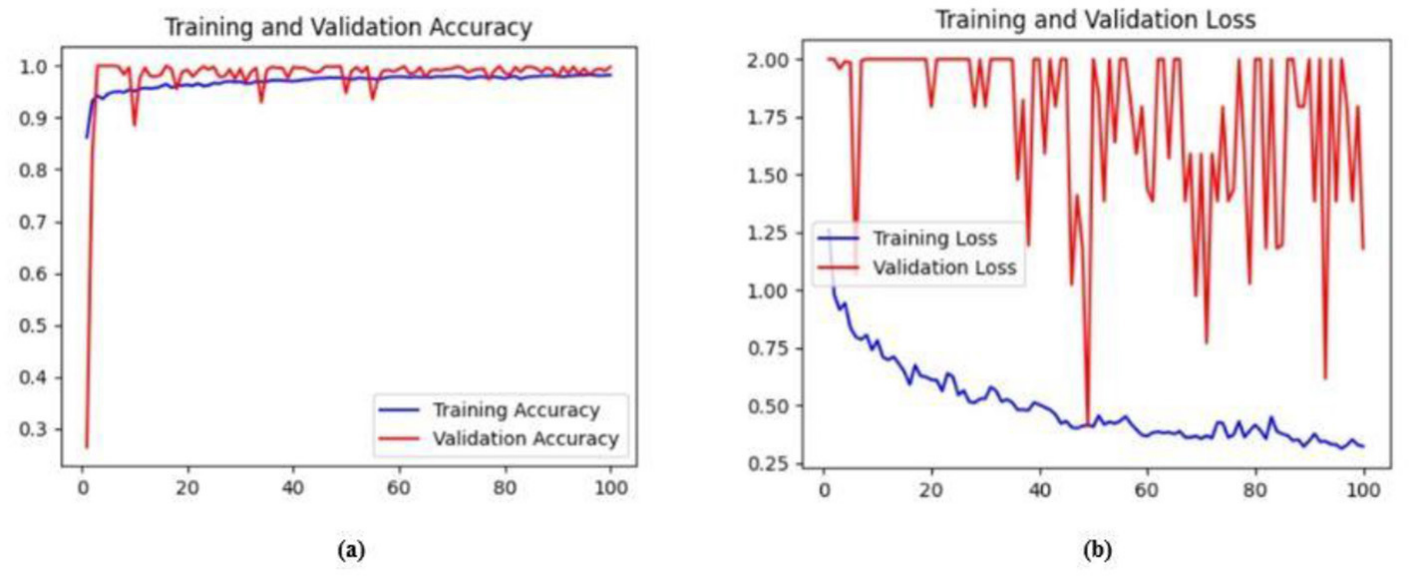
**(a)** Training and validation accuracy of Model 3 on the BUSI dataset. **(b)** Training and validation loss of Model 3 on the BUSI dataset.

**Figure 16:**
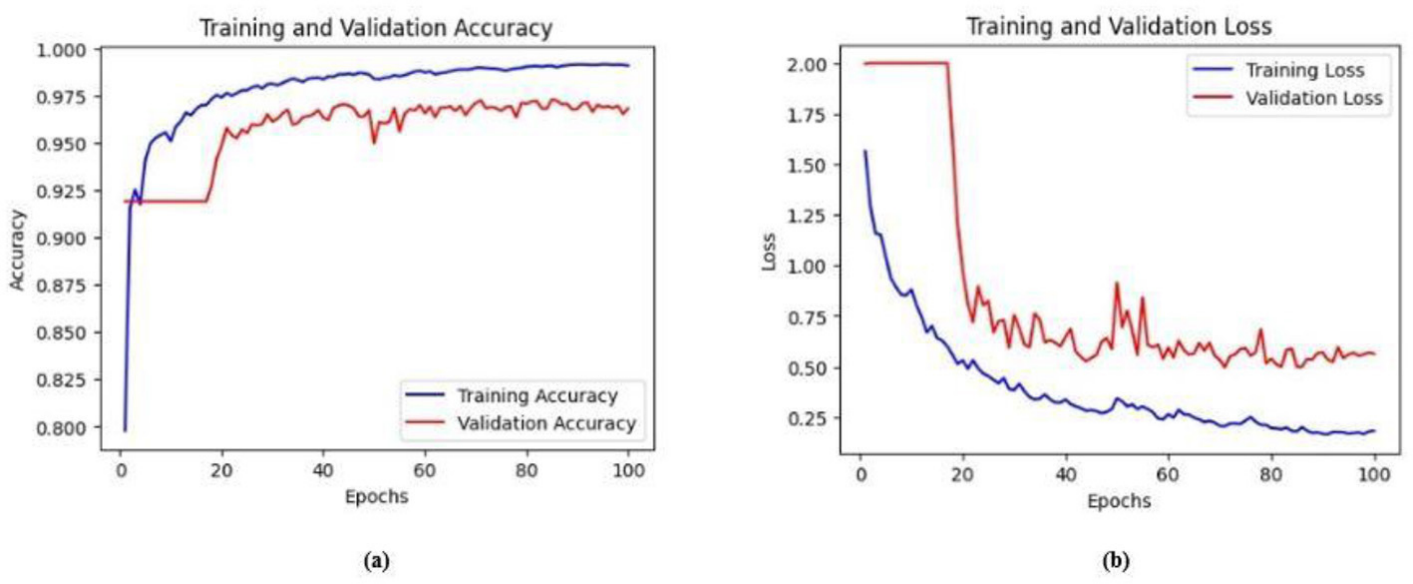
**(a)** Training and validation accuracy of Model 3 on the BrEaST dataset. **(b)** Training and validation loss of Model 3 on the BrEaST dataset.

**Figure 17:**
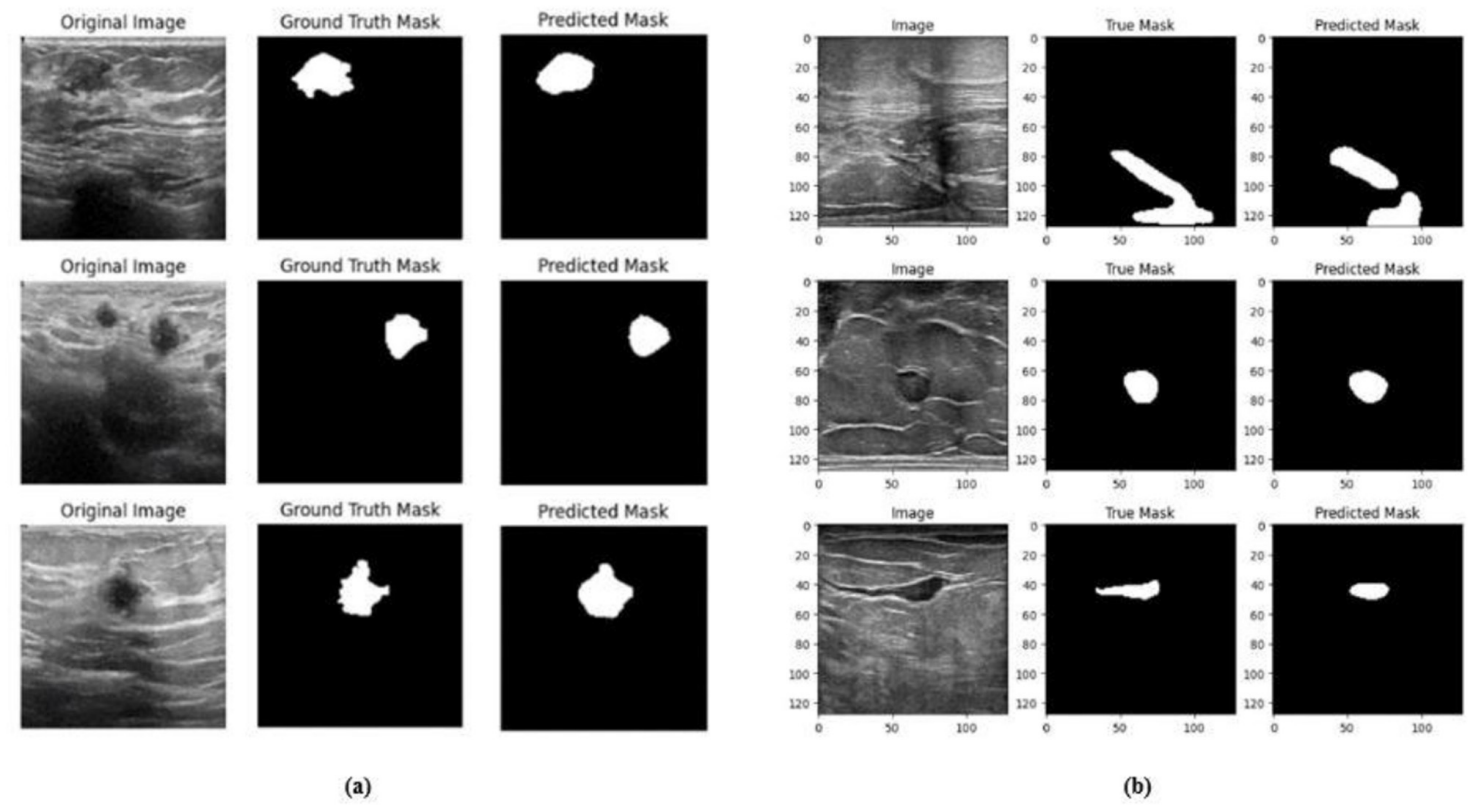
Visualizations of the segmentation results of Model 3 on the **(a)** BUSI dataset and **(b)** BrEaST dataset.

Ablation studies were implemented to investigate the impact of enhanced ConvMixer (*ECM*), convolution-enhanced multihead attention (*CE*_*MHA*_) and the enhanced ConvNeXT (*ECN*) modules on the segmentation of breast ultrasound images. The basic model without ConvMixer and ConvNeXT modules (Model 1) was initially implemented. ECMs were integrated next (Model 2) to assess its impact on the performance of the model. The next enhancement was to integrate enhanced ConvNeXT (Model 3), followed by the addition of a convolution-enhanced multihead attention module (HMA-Net). The efficiency of each model was evaluated on the BUSI and BrEaST datasets using the Jaccard index, DSC, accuracy, precision, and recall. Training and validation accuracy and loss curves for each model (Figures 9, 10 for Model 1, Figures 11, 12 for Model 2, and Figures 13, 14 for Model 3) along with visualizations of segmentations (Figures 15a, b for Model 1, Figures 16a, b for Model 2, and Figures 17a, b for Model 3) are also presented, demonstrating the incremental enhancement in segmentation performance across all the stages.

#### 4.5.1 Performance analysis of model 1 (base model)

The base model has an encoder–decoder structure with symmetrical layers that perform downsampling and upsampling operations on the input image, respectively. The encoder section consists of five blocks, each consisting of a convolutional layer, batch normalization and GeLU activation. Downsampling is accomplished by employing max pooling layers. The bottleneck utilizes a simple structure that incorporates additional convolution and normalization layers. The decoder section of the model uses Conv2DTranspose layers to increase the resolution of the feature maps. This base model, without the use of ConvMixer, ConvNeXT, and a simple bottleneck with convolutions (instead of multihead attention), attained a Jaccard index of 50.47% for the BUSI dataset, and for the BrEaST dataset, the Jaccard index was 44.71%. The DSCs obtained were 67.08% for the BUSI dataset and 61.79% for the BrEaST dataset. The accuracy, precision, and recall values obtained were 95.69%, 84.29%, and 55.71% for the BUSI dataset, and 96.13%, 89.69%, and 47.13% for the BrEaST dataset, respectively. The training and validation accuracy for Model 1 on the BUSI dataset is shown in Figure 9a, and the training and validation loss is displayed in Figure 9b. The training and validation accuracy for Model 1 on the BrEaST dataset is displayed in Figure 10a, and the training and validation loss is displayed in Figure 10b. The visualizations of the segmentation results for Model 1 on the BUSI dataset are shown in Figure 11a, while the results for the BrEaST dataset are shown in Figure 11b.

#### 4.5.2 Performance analysis of model 2 (enhanced ConvMixer modules integrated with the base model)

Enhanced ConvMixer modules were added to the encoder blocks of Model 1, significantly improving the ability of the model to capture spatial patterns. It allowed the model to effectively integrate spatial and channel information, enabling it to focus on relevant features. The integration of ECM modules also reduced the risk of vanishing gradient problems, resulting in effective training and improved performance. For the BUSI dataset, the model obtained a Jaccard index of 83.22%, a DSC of 90.84%, an accuracy of 98.52%, a precision of 91.97%, and a recall of 88.52%. For the BrEaST dataset, the Jaccard index was 80.57%, the DSC was 89.24%, the accuracy was 98.60%, the precision was 91.28%, and the recall was 87.30%.

The training and validation accuracy of Model 2 on the BUSI dataset is shown in Figure 12a, and the training and validation loss is displayed in Figure 12b. The training and validation accuracy of Model 2 on the BrEaST dataset is shown in Figure 13a, and the training and validation loss is shown in Figure 13b. The visualizations of the segmentation results obtained using Model 2 on the BUSI dataset are shown in Figure 14a, while those for the BrEaST dataset are presented in Figure 14b.

#### 4.5.3 Performance analysis of model 3 (enhanced ConvNeXT modules integrated with model 2)

The next enhancement made was to integrate enhanced ConvNeXT (ECN) modules into the five contiguous decoder blocks of Model 2. It improves the feature representation ability of the model, and high-resolution segmentation maps can be generated by combining transposed convolutions with ConvNeXT. The model achieved a Jaccard index of 91.79%, a DSC of 95.72%, an accuracy of 99.32%, a precision of 93.36%, and a recall of 98.20% on the BUSI dataset. For the BrEaST dataset, the Jaccard index was 85.08%, the DSC was 91.94%, the accuracy was 98.94%, the precision was 93.28%, and the recall was 90.64%. The training and validation accuracy of Model 3 on the BUSI dataset is shown in Figure 15a, and the training and validation loss is displayed in Figure 15b. Similarly, the training and validation accuracy of Model 3 on the BrEaST dataset is displayed in Figure 16a, and the training and validation loss is shown in Figure 16b. The visualizations of the segmentation results of Model 3 on the BUSI dataset are displayed in Figure 17a, while the results obtained with the BrEaST dataset are shown in Figure 17b.

The Jaccard index, DSC, recall, accuracy, and precision obtained for Model 1, Model 2, and Model 3 are displayed in Table 2. Model 1 (the base model) obtained lower Jaccard and Dice scores, demonstrating its limited ability for precise segmentation. Visualization results show that the lesion boundaries are poorly defined due to the restricted expressive power of conventional convolutional layers in the encoder and decoder. The performance was enhanced with the addition of ECMs in Model 2, which utilized depthwise and pointwise convolutions for reduced computational complexity, in conjunction with squeeze and excitation to improve the integration of spatial and channel features. The model obtained higher Dice scores of 90.84% on the BUSI dataset and 89.24% on the BrEaST dataset, demonstrating improved generalization. Visualization of the segmentation results also indicates clearer tumor contours. Enhanced ConvNeXT modules were integrated within the decoder, resulting in Model 3. The ECN blocks enhanced the upsampled representations through depthwise convolution, channel mixing, and recalibration utilizing *S*_E_ layers. The squeeze and excitation layer in the encoder and decoder reduces the parameter overhead while enhancing feature selection. High-resolution masks were reconstructed with sharper boundaries and reduced false positives. Intricate semantic information can be retrieved as indicated by a recall value of 98.20% on the BUSI dataset and 90.64% on the BrEaST dataset.

**Table 2:** Performance analysis of model 1, model 2, and model 3.

**Model**	**Jaccard index (%)**	**Dice similarity coefficient (%)**	**Recall (%)**	**Accuracy (%)**	**Precision (%)**
**BUSI dataset**					
Model 1	50.47	67.08	55.71	95.69	84.29
Model 2	83.22	90.84	88.52	98.52	91.97
Model 3	91.79	95.72	98.20	99.32	93.36
**BrEaST dataset**					
Model 1	44.71	61.79	47.13	96.13	89.69
Model 2	80.57	89.24	87.30	98.60	91.28
Model 3	85.08	91.94	90.64	98.94	93.28

## 5 Experimental results and performance analysis of proposed HMA-Net

A detailed analysis of the proposed hybrid mixer framework with multihead attention for breast ultrasound image segmentation (HMA-Net) is carried out in this session. The HMA-Net utilized *EM*_*x*_*i*__ blocks with enhanced ConvMixer for capturing multiscale features at varying stages from the input ultrasound images and is converted into a sequence of more detailed and compact representations with reduced resolutions, which in turn is used by the *DCN*_*x*_*i*__ blocks to perform effective segmentations. The spatial resolutions of the extracted feature maps are restored to the original level of the input image by the *DCN*_*x*_*i*__ blocks using transposed convolutions and enhanced ConvNeXT modules. The *CE*_*MHA*_ module with normalized convolutions and multihead attention with residual connections escalates the feature extraction property of HMA-Net by allowing the model to concentrate more on relevant features. The convolution-enhanced multihead attention allows the capture of contextual dependencies between various components of the image, thus empowering the model to differentiate minute differences in the breast ultrasound images. The implementation of spatial attention at the bottleneck *via* *CE*_*MHA*_ avoids the need for spatial attention across all the layers, thereby reducing computational complexity while capturing global contextual dependencies.

For the BUSI dataset, the model achieved a Jaccard index of 98.04% and a DSC of 99.01%. The model is efficient in detecting actual tumor regions, indicated by a recall of 99.09%. Precision and accuracy obtained were 99.06% and 99.85%, respectively. This is important in medical diagnosis to prevent missed detections. The values obtained indicate the ability of the model to correctly identify both tumor and non-tumor regions. The results obtained for the BrEaST dataset are as follows. The model achieved a Jaccard index of 94.84% and a DSC of 97.35%. The model can correctly identify tumor and non-tumor regions, as demonstrated by an accuracy of 99.65% and a precision of 98.67%. The recall achieved was 96.03%. In summary, based on the evaluation of the two datasets, these findings indicate that the model has significant potential for clinical use in precisely delineating breast tumors from ultrasound images, hence assisting in the timely identification and diagnosis of breast cancer. The summary of the results obtained is shown in Table 3.

**Table 3:** Results obtained by the HMA-Net for the two different datasets.

**Performance metrics**	**BUSI dataset**	**BrEaST dataset**
Jaccard index	98.04%	94.84%
Dice similarity coefficient	99.01%	97.35%
Recall	99.09%	96.03%
Accuracy	99.85%	99.65%
Precision	99.06%	98.67%

**Figure 18:**
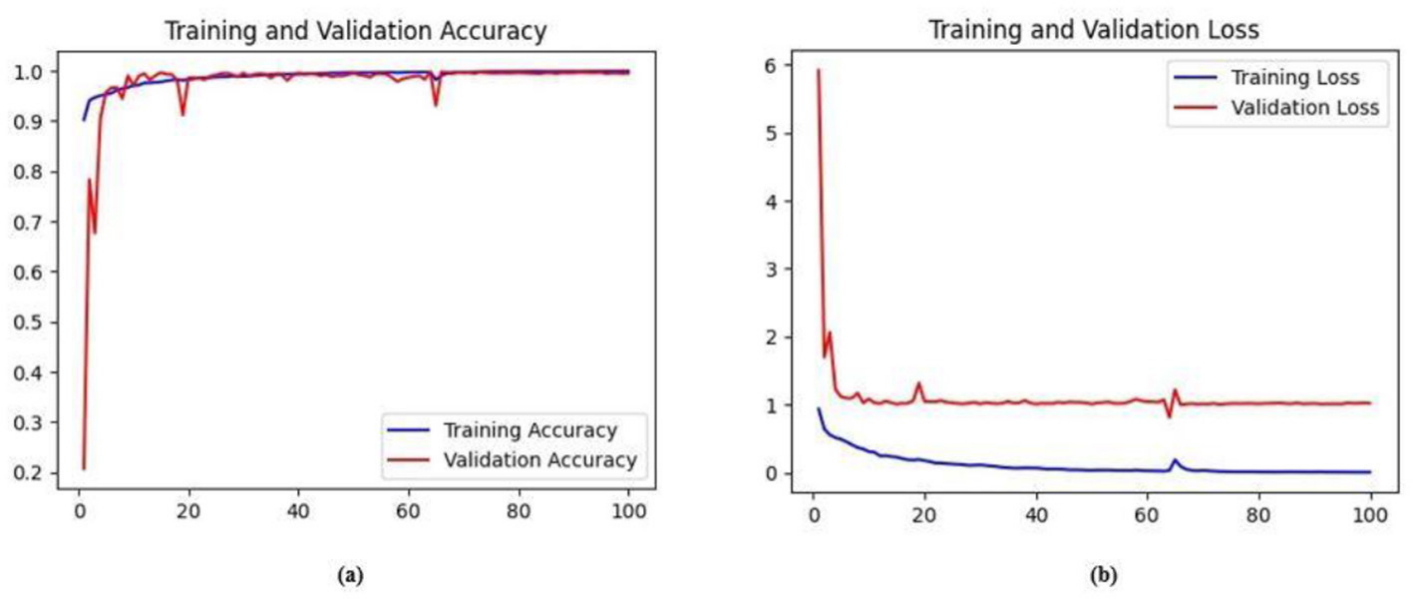
**(a)** Training and validation accuracy of the HMA-Net on the BUSI dataset. **(b)** Training loss and validation loss of the HMA-Net on the BUSI dataset.

**Figure 19:**
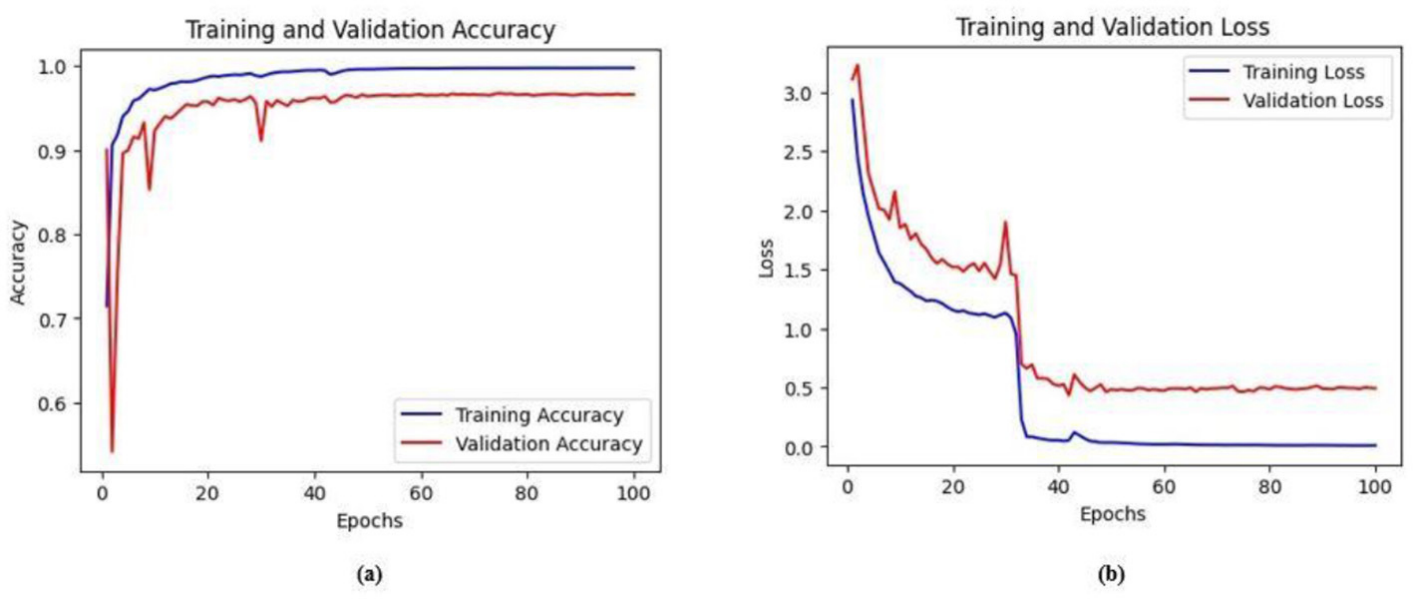
**(a)** Training and validation accuracy of the HMA-Net on the BrEaST dataset. **(b)** Training and validation loss of the HMA-Net on the BrEaST dataset.

**Figure 20:**
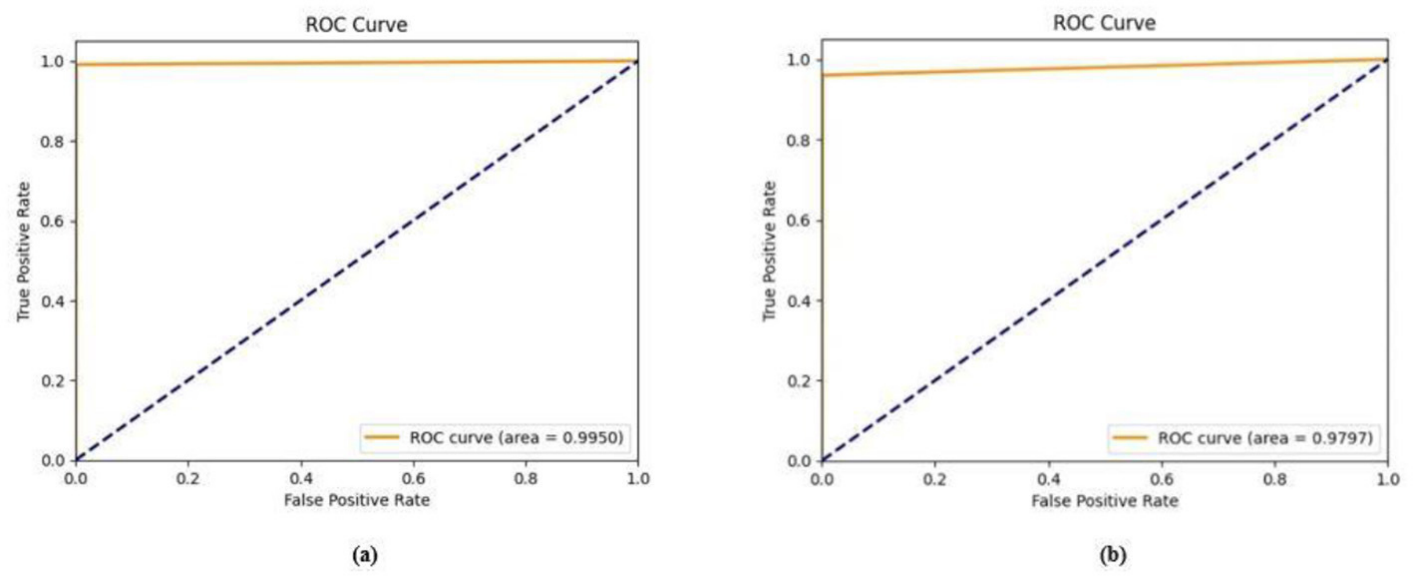
AUC curve obtained for the **(a)** BUSI dataset and **(b)** BrEaST dataset.

**Figure 21:**
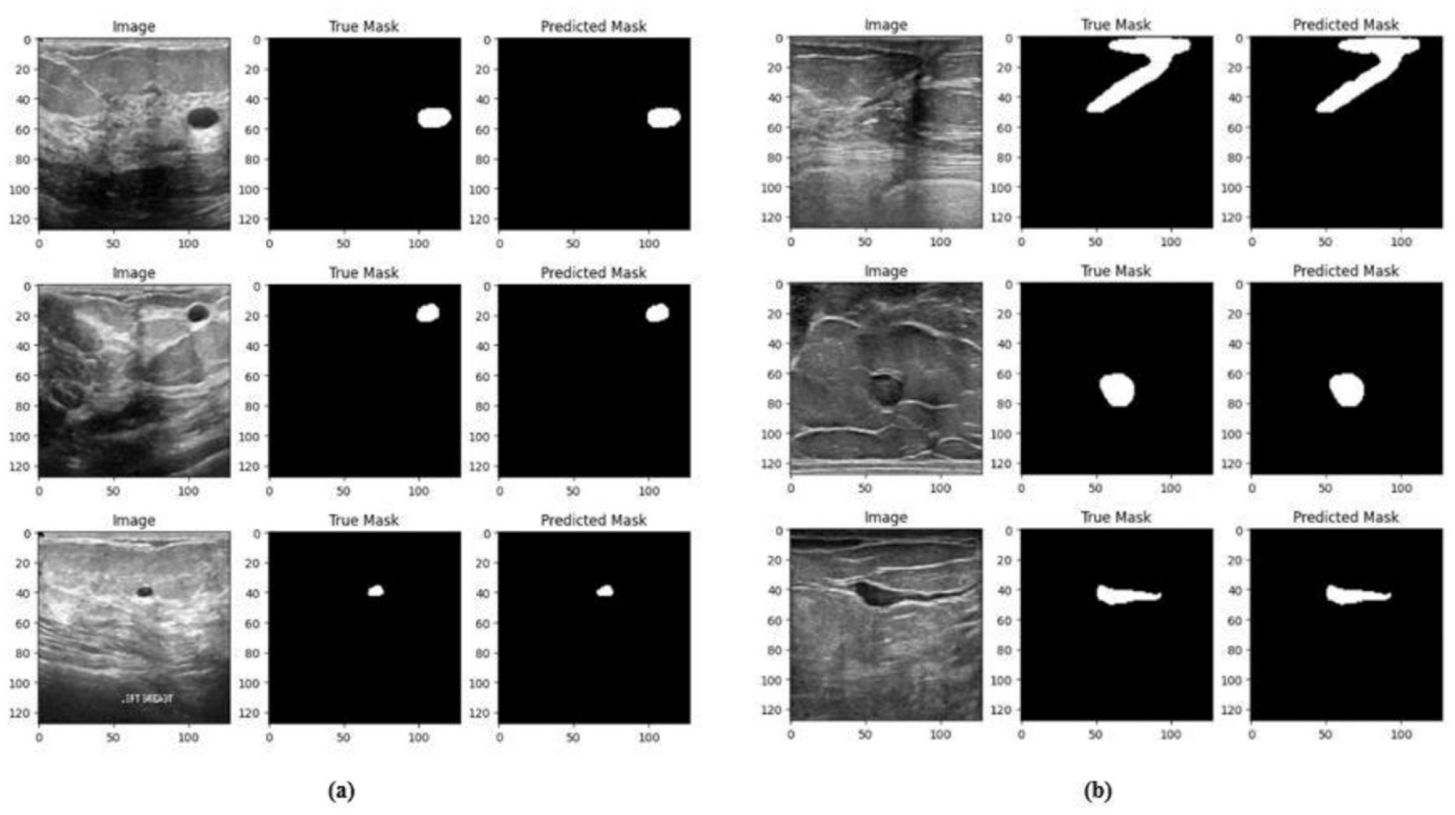
Visualization of the segmentation results of HMA-Net on **(a)** BUSI dataset and **(b)** BrEaST dataset.

The training and validation accuracy obtained for the proposed HMA-Net on the BUSI dataset is displayed in Figure 18a, and the training and validation loss obtained is displayed in Figure 18b. Figure 19a displays the training and validation accuracy obtained for the HMA-Net on the BrEaST dataset, and the training and validation loss for the BrEaST dataset is shown in Figure 19b. HMA-Net obtained AUC values of 0.9950 for the BUSI dataset and 0.9797 for the BrEaST dataset. These values indicate that the model is highly effective in differentiating background pixels from tumor regions. The AUC curve obtained for the BUSI dataset is displayed in Figure 20a, and the BrEaST dataset is presented in Figure 20b. The visualizations of the segmentation results obtained for the BUSI dataset are displayed in Figure 21a, while those for the BrEaST dataset are displayed in Figure 21b.

The results obtained in Table 3 for the BUSI and BrEaST demonstrate that the proposed HMA-Net can be effectively used for the segmentation of tumor regions. The performance of the HMA-Net is due to the combined contribution of various components—ECMs in the encoder, enhanced ConvNeXT modules (ECN) in the decoder, and convolution-enhanced multihead attention (*CE*_*MHA*_) at the bottleneck. The ECM blocks improve spatial feature extraction using depthwise and pointwise convolutions, utilizing *S*_E_ techniques for adaptive channel recalibration, enabling the model to prioritize relevant features and diminish noise. The *ECN* blocks further enhance the upsampled features, guaranteeing precise reconstruction of segmentation masks with more defined lesion boundaries. The local and global feature representations are merged using *CE*_*MHA*_ module by integrating convolutional processing and multihead attention, thereby allowing the network to capture long-range relationships and contextual information essential for accurate tumor localization.

## 6 Comparison of the HMA-Net with the state-of-the-art architectures

Table 4 provides a comparison between the HMA-Net and the other models. The method introduced by Üzen (2024) obtained a Jaccard index of 69.23% and a DSC of 80.23% on the BUSI dataset. Zhang et al. (2023) introduced a method for breast ultrasound image classification and segmentation and obtained a DSC of 89.8%, a Jaccard index of 79.1% and a recall of 85.9%. The regional attentive multitask learning framework proposed by Xu et al. (2023) was evaluated on two datasets, obtaining DSC values of 85.69% and 80.04%, sensitivity values of 89.51% and 82.54%, specificity values of 99.25% and 98.00%, accuracy values of 98.79% and 96.4%, and IOU values of 77.84% and 71.93%. The method introduced by Chen et al. (2023) was evaluated on two different datasets to obtain Jaccard index values of 70.36% and 73.17%, DSC values of 78.51% and 81.50%, specificity values of 97.42% and 99.05%, precision values of 79.73% and 82.58%, and recall values of 82.70% and 84.02%. The method proposed by Lyu et al. (2023) was also evaluated using two datasets with the DSC values of 80.71% and 79.62%, specificity values of 98.54% and 99.38%, accuracy values of 97.13% and 97.97%, precision values of 83.5% and 87.95%, recall values of 79.3% and 74.43%, and IOU values of 68.53% and 67.52%. The method proposed by Almajalid et al. (2018) secured a DSC of 82.52%, a TPR of 78.66%, an FPR of 18.59%, and a FNR of 21.34%.

**Table 4:** Comparison of the HMA-Net with the state-of-the-art architectures.

**References**	**Dataset**	**Jaccard index (%)**	**Dice similarity coefficient (%)**	**Recall (%)**	**Accuracy (%)**	**Precision (%)**
Üzen (2024)	BUSI	69.23	80.23			
Zhang et al. (2023)	1,600 breast ultrasound images	0.791	0.898	85.9		
Xu et al. (2023)	UDIAT	77.84	85.69	89.51	98.79	
	BUSI	71.93	80.04	82.54	96.4	
Chen et al. (2023)	BUSI	70.36	78.51	82.70		79.73
	Dataset B	73.17	81.50	84.02		82.58
Lyu et al. (2023)	BUSI	68.53	80.71	79.30	97.13	83.5
	OASBUD	67.52	79.62	74.43	97.97	87.92
Almajalid et al. (2018)	221 breast ultrasound images		82.52	78.66		
Cho et al. (2022)	BUSI	77.835	84.856		97.253	
	UDIAT	77.094	85.366		98.601	
Vakanski et al. (2020)	Dataset of 510 breast ultrasound images	0.838	0.905	0.910	0.980	
Tang et al. (2023)	BUSI	73.27%		84.26	97.33	84.81
Huang et al. (2021)	Dataset with 325 breast ultrasound images	81.29		90.33		
Ilesanmi et al. (2021)	Dataset with 264 images		89.73			
Tong et al. (2021)	Dataset with 830 images		0.959	0.979	0.850	
AbdElhakem and Torki (2023)	Dataset of 316 breast ultrasound images	68.17	80.60			
Shareef et al. (2022)	BUSI	0.70	0.78	0.80		
	BUSIS	0.86	0.92	0.91		
	Dataset B	0.74	0.82	0.84		
He et al. (2023)	BUSI	71.84	82	82.14	96.94	83.24
	BUS	73.83	84.13	83.19	98.49	88.50
	Dataset B	94.63	97.23	97.33	97.41	97.14
Zhang et al. (2024)	UDIAT		88.73 ± 2.11		99.03 ± 0.32	88.68 ± 2.25
	BLUI		89.48 ± 0.44		96.96 ±0.42	89.93 ± 1.15
	BUSI		83.11 ± 2.07		96.80 ±0.16	86.08 ± 2.52
Zhai et al. (2022)	DBUI		0.8690		0.9760	
	SPDBUI		0.9391		0.9508	
	ADBUI		0.7644		0.9605	
	SDBUI		0.8319		0.9589	
Lin et al. (2023)	BUSI Benign		0.8127 ± 0.2178			0.7932 ± 0.2382
	BUSI Malignant		0.6939 ± 0.2401			0.6943 ± 0.2594
	MT_BUS		0.8016 ± 0.1722			0.8021 ± 0.1976
	BUL		0.8698 ± 0.1200			0.8938 ± 0.1263
HMA-Net	BUSI	**98.04**	**99.01**	**99.09**	**99.85**	**99.06**
	BrEaST	**94.84**	**97.35**	**96.03**	**99.65**	**98.67**

Bolded values represent the performance of the proposed method (HMA-Net).

The method proposed by Cho et al. (2022) obtained a pixel accuracy of 97.253%, an IOU of 77.835%, and a Dice coefficient of 84.856% on the BUSI dataset. For the UDIAT dataset, the same method achieved a pixel accuracy of 98.601%, an IOU of 77.094%, and a Dice coefficient of 85.366%. Attention blocks enhanced U-Net architecture proposed by Vakanski et al. (2020) attained a Jaccard index of 83.8%, a DSC of 90.5%, a TPR of 91.0%, an FPR of 8.9%, and an accuracy of 98.0%. The ConvMixer-based model for ultrasound image segmentation proposed by Tang et al. (2023) obtained an IOU of 84.75 ± 0.30, a recall of 91.53 ± 0.37, a precision of 92.02±0.13, an F1 score of 84.16 ± 0.47 and an accuracy of 97.33 ± 0.14 on the BUSI dataset. An IOU of 81.29%, an FPR of 9.00%, and a Recall of 90.33% were obtained for fuzzy deep learning network-based breast ultrasound image segmentation proposed by Huang et al. (2021). The method proposed by Ilesanmi et al. (2021) obtained a Dice measure of 89.73%. The method proposed by Tong et al. (2021) obtained a Dice coefficient of 95.9%, a sensitivity of 97.9%, an accuracy of 85%, and a specificity of 92.8%.

The method proposed by AbdElhakem and Torki (2023) secured an IOU of 68.17% and a Dice score of 80.60%. The model introduced by Shareef et al. (2022) was evaluated on three different datasets, obtaining Jaccard index values of 70%, 86%, and 74%, DSC values of 78%, 92%, and 82%, TPR values of 80%, 91% and 84%, and FPR values of 36%, 7%, and 22%, respectively. The hybrid convolutional neural network proposed by He et al. (2023) was also evaluated on three different datasets, achieving Jaccard index values of 71.84%, 73.83%, and 94.63%, DSCs of 82%, 84.13%, and 97.23%, accuracy values of 96.94%, 98.49%, and 97.41%, precision values of 83.24%, 88.50%, and 97.14%, and recall values of 82.14%, 83.19%, and 97.33, respectively. The model proposed by Zhang et al. (2024) obtained a Dice coefficient of 88.73% for the UDIAT dataset, 89.48% for the BLUI dataset, and 83.11% for the BUSI dataset, while the corresponding accuracy values were 99.03 ± 0.32, 96.96 ± 0.42, and 96.80 ± 0.16.

The method proposed by Zhai et al. (2022) obtained DSC of 0.8690, 0.9391, 0.7644, and 0.8319 on DBUI, SPDBU, ADBUI, and SDBUI datasets, respectively. The corresponding accuracy values of these datasets were 0.9760, 0.9508, 0.9605, and 0.9589. The method proposed by Lin et al. (2023) obtained DSCs of 0.8127 ± 0.2178, 0.6939 ± 0.2401, 0.8016 ± 0.1722, and 0.8698 ± 0.1200 on the BUSI benign, BUSI malignant, MT_BUS, and BUL datasets, respectively. In contrast, the precision values obtained were 0.7932 ± 0.2382, 0.6943 ± 0.2594, 0.8021 ± 0.1976, and 0.8938 ± 0.1263 for the same datasets.

The HMA-Net model obtained better results than prior techniques, such as U-Net, U-Net++, and ConvMixer-based architectures, by combining global and local feature extraction. The model utilized *EM*_*x*_*i*__ blocks integrated with ECMs to capture varied characteristics through depthwise and pointwise convolutions, while squeeze-and-excitation (*S*_E_) recalibrate channel-wise responses for improved feature representation. Enhanced ConvNeXT based *DCN*_*x*_*i*__ blocks further refine the upsampled features by integrating residual connections and channel mixing, facilitating the precise reconstruction of segmentation masks.

Unlike previous architectures that prioritize either local patterns or long-range dependencies, HMA-Net effectively combines both with its convolution-enhanced multihead attention (CE_MHA_) module, which merges convolutional operations for local feature extraction with global attention. This design enhances the delineation of ambiguous tumor boundaries in ultrasound images. Integrating attention exclusively at the bottleneck stage, rather than throughout all layers, achieves an optimal equilibrium between performance and efficiency. These distinct components jointly enhance the segmentation outcomes attained by HMA-Net on both the BUSI and BrEaST datasets, as reflected in Table 4.

## 7 Conclusion

Breast cancer is a prevalent issue among women nowadays and is impacting the lives of numerous individuals. Ultrasound images have now been widely used for detecting breast cancer owing to their safe and radiation-less nature. A hybrid mixer framework with multihead attention (HMA-Net) has been proposed for segmenting breast ultrasound images. The HMA-Net utilizes enhanced ConvMixer-based *EM*_*x*_*i*__ blocks for extracting downsampled feature maps from the input ultrasound images, and high-resolution segmentation masks are reconstructed using enhanced ConvNeXT-based *DCN*_*x*_*i*__ blocks. The ability of ECMs to combine the channel and spatial information is enhanced with the addition of the squeeze and excitation layer by dynamically adjusting the importance of various channels, resulting in more discriminative and detailed feature representations. The enhanced ConvNeXT modules capture complex patterns and hierarchical characteristics by which high-resolution segmentation masks can be reconstructed from low-resolution encoded features. The variations in the input data can be handled by ConvNeXT modules, and more accurate segmentation masks can be constructed by capturing local and global features. The residual linking improves the performance of the model by enhancing feature propagation and maintaining important features across layers. The convolution-enhanced multihead attention module improves the performance of the model by capturing long-range dependencies and intricate patterns from the input images. The model utilized a combined loss function, which enables the model to handle unbalanced data and to concentrate more on relevant areas. The performance of the model was evaluated using two breast ultrasound image datasets. The model obtained a Jaccard index of 98.04% and a DSC of 99.01% on the BUSI dataset. For the BrEaST dataset, the model obtained a Jaccard index of 94.84% and a DSC of 97.35%. The results obtained indicate that the model can be efficiently used for segmenting breast ultrasound images, which will help in the early detection of breast cancer. The HMA-Net model has robust segmentation capabilities and may be effortlessly incorporated into current ultrasound imaging workflows for clinical use. A practical scenario involves direct real-time implementation on ultrasound scanners and generating instantaneous segmentation masks during image acquisition. It can also be implanted in post-diagnostic systems to analyze the captured images and generate segmentation outputs prior to radiologist evaluation. This facilitates better lesion analysis, quicker interpretation, and consistent reporting in large-scale clinical environments.

The HMA-Net model has been evaluated on the BUSI and the BrEaST datasets. The BUSI dataset consists of 780 images collected from a single hospital, while the BrEaST dataset contains 256 manually annotated scans collected from five different institutions. Since these datasets have limited size and diversity, the variations present in real-world clinical situations cannot be adequately represented by this. Bigger datasets with a wider variety of image qualities, scanner types, and patient demographics should be used to validate the robustness and scalability of the proposed methodology.

## Data Availability

The original contributions presented in the study are included in the article/supplementary material, further inquiries can be directed to the corresponding author.
